# IGF2BP3 enhances lipid metabolism in cervical cancer by upregulating the expression of SCD

**DOI:** 10.1038/s41419-024-06520-0

**Published:** 2024-02-14

**Authors:** Chenying Han, Chenchen Hu, Tianyue Liu, Yuanjie Sun, Feiming Hu, Yuanli He, Jiaxing Zhang, Jiaxi Chen, Jiaqi Ding, Jiangjiang Fan, Xiyang Zhang, Jing Wang, Xupeng Qiao, Dongbo Jiang, Kun Yang, Shuya Yang

**Affiliations:** 1 Department of Immunology, the Fourth Military Medical University, 710032 Xi’an, Shaanxi China; 2 Department of Dermatology, Xijing Hospital, Fourth Military Medical University, 710032 Xi’an, Shaanxi China; 3 Department of Neurology, Tangdu Hospital, Fourth Military Medical University, 710038 Xi’an, Shaanxi China; 4 Department of Thoracic Surgery, Tangdu Hospital, Fourth Military Medical University, 710038 Xi’an, China; 5 Military Medical Innovation Center, the Fourth Military Medical University, 710032 Xi’an, Shaanxi China; 6 Department of Rheumatology and Immunology, Tangdu Hospital of the Air Force Medical University, 710038 Xi’an, Shaanxi China

**Keywords:** Cancer metabolism, Tumour biomarkers

## Abstract

Cervical cancer (CC) is the most common gynecologic malignancy, which seriously threatens the health of women. Lipid metabolism is necessary for tumor proliferation and metastasis. However, the molecular mechanism of the relationship between CC and lipid metabolism remains poorly defined. We revealed the expression of IGF2BP3 in CC exceeded adjacent tissues, and was positively associated with tumor stage using human CC tissue microarrays. The Cell Counting Kit-8, colony formation assay, 5-ethynyl-2′-deoxyuridine assay, transwell assays, wound-healing assays, and flow cytometry assessed the role of IGF2BP3 in proliferation and metastasis of CC cells. Besides, exploring the molecular mechanism participating in IGF2BP3-driven lipid metabolism used RNA-seq, which determined SCD as the target of IGF2BP3. Further, lipid droplets, cellular triglyceride (TG) contents, and fatty acids were accessed to discover that IGF2BP3 can enhance lipid metabolism in CC. Moreover, RIP assay and methylated RNA immunoprecipitation experiments seeked the aimed-gene-binding specificity. Lastly, the IGF2BP3 knockdown restrained CC growth and lipid metabolism, after which SCD overexpression rescued the influence in vitro and in vivo using nude mouse tumor-bearing model. Mechanistically, IGF2BP3 regulated SCD mRNA m6A modifications via IGF2BP3-METTL14 complex, thereby enhanced CC proliferation, metastasis, and lipid metabolism. Our study highlights IGF2BP3 plays a crucial role in CC progression and represents a therapeutic latent strategy. It is a potential tactic that blocks the metabolic pathway relevant to IGF2BP3 with the purpose of treating CC.

## Introduction

Cervical cancer embodies a large part of the cancer burden of women in the world [1], which is the fourth consecutively diagnosed cancer and the fourth leading reason of cancer death in women on the basis of Global assessment in 2020 [2]. Cervical cancer is the most common gynecological malignant tumor, which occupies the first place in female reproductive organ tumors. The latest research results show that there are 570,000 new cases of cervical cancer and 310,000 deaths worldwide every year [3]. According to the annual report of China Cancer Registry, there are 150,000 new cases of cervical cancer in China every year, accounting for 73-93% of the incidence of malignant tumors in female reproductive system. Especially in recent years, the incidence of cervical cancer among young people is increasing obviously, with an annual rate of 2–3% [4], which seriously threatens the health of Chinese women. CC-associated death rate is still high principally because of its undefined nosogenesis. Therefore, identification of fresh therapeutical target spot is hopeful to go a step further comprehend the CC nosogenesis. RNA binding proteins (RBPs) as a family of proteins specifically binding to RNAs, participate in post-transcriptional regulating of gene expression and important cell differentiation and metabolism on many aspects [5]. RBPs are the pivotal effect factors of gene expression, RNA metabolized modulated in various aspects, and form a broad regulatory network to help maintain cell homeostasis [6]. RBP dysregulation leads to unusual phenotypes of protein, then gives rise to cancers happening, growing, and metabolism-associated molecular activity [7]. RBPs-mediated regulation is of vital importance in mechanotransduction promoting cancer metastasis [8]. IGF2BP3, as a RBP, may enhance cancer-derived cellular growth and migratory ability in tumor progression [9].

Tumor metabolism, such as high rate of de novo fatty acid synthesis, meets the energetic and biosynthetic requirements of quickly proliferative cancerous cells and alters intracellular and intercellular signal transduction, thereby promoting invasion, metastasis, and immune escape in tumors [10]. Lipid metabolism is associated with the developing process, including ECM remolding, shifting in the interplay between cell and ECM, and reorganization of cytoskeleton, of various cancers consist of breast cancer, endometrial cancer, esophageal cancer, etc. [11]. Malignant tumors overexpress SCD, including lung cancer, breast cancer, colorectal cancer, esophageal cancer, bladder cancer, and liver cancer. Stearoyl-CoA desaturase (SCD) converted saturated fatty acids (SFAs) into unsaturated fatty acids (UFAs), which are part of fatty acids, an important source of energy in tumor cells. Even though the therapeutic target spot of lipid metabolism correlative elements would change tumor behavior and restrain cancer growth, the related molecular mechanisms are not clear.

Up to now, IGF2BP3 function still does not receive system study in CC. During this research, the immunohistochemical (IHC) staining announced that compared with adjacent normal tissues, IGF2BP3 expression was obviously higher in CC tissues and it is a positive correlation between high IGF2BP3 expression and cervical cancer staging. Furthermore, we discover that IGF2BP3 knockdown in CC cells eminently weakens proliferation, metastasis, and lipid metabolism in vitro and in vivo. Besides, RNA-sequencing (RNA-seq) results revealed that SCD expressed strikingly lower in IGF2BP3-knockout cells. In addition, It was demonstrated that SCD overexpression in IGF2BP3-knockout CC cells can rescued the ability of proliferation, metastasis and lipid metabolism. Mechanistically, IGF2BP3 binding to SCD via N6-methyladenosine (m6A) modification regulates the lipid metabolism pathway through affecting the key enzymes activity. On the whole, our study elucidated that IGF2BP3 functions in CC oncogenesis and progression and provides a new clinical treatment tactics to cervical cancer patients.

## Results

### High expression of IGF2BP3 in cervical cancer is related to tumor stage

**Fig. 1:**
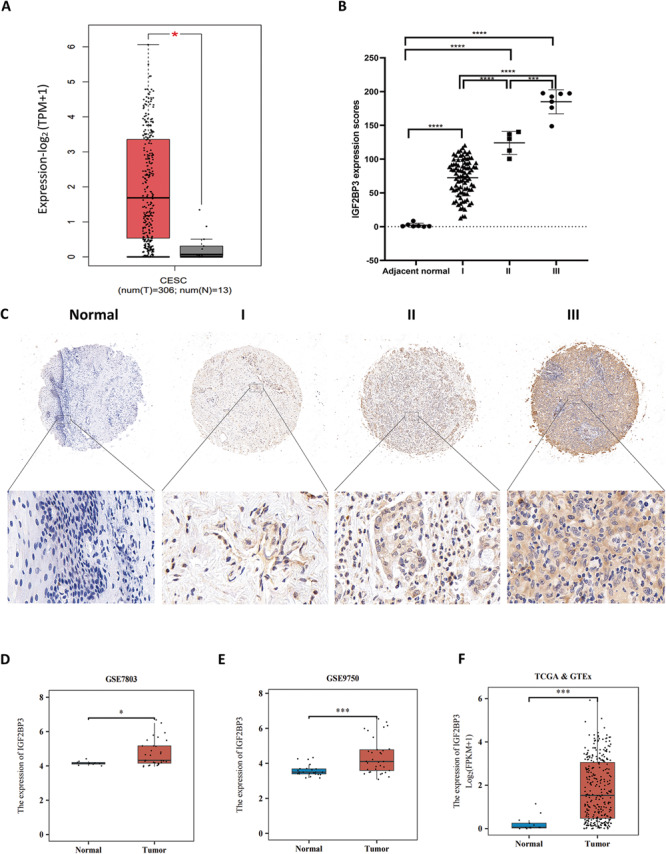
High expression of IGF2BP3 in cervical cancer is related to tumor stage. **A** GEPIA database (http://gepia.cancer-pku.cn/detail.php) was used to analyze the difference of IGF2BP3 expression between CC tissues and normal tissues. **B** The expression of IGF2BP3 in different stages of CC and adjacent normal tissues (*n* = 108; Student’s *t* test). **C** The expression of IGF2BP3 in para-carcinoma tissues and CC tumor tissues at various clinical stages examined by IHC analysis. **D**–**F** Differences in IGF2BP3 expression between CC tissue and normal tissue were analyzed using CESC microarrays (GSE7803、GSE9750 and TCGA&GETx). *P < 0.05, ***P < 0.001, and ****P < 0.0001.

In order to define the function of IGF2BP3 in CC, the difference in IGF2BP3 expression between CC tissues and normal tissues from the Gene Expression Profiling Interactive Analysis (GEPIA) database (http://gepia.cancer-pku.cn/detail.php) were analyzed (Fig. 1A). Then IHC staining of IGF2BP3 in 108 patients tissues comparing with adjacent normal tissues showed that IGF2BP3 expression was obviously higher in CC tissues and IGF2BP3 expression was directly related to CC stage (Fig. 1B, C). High expression of IGF2BP3 in cervical cancer has a positive correlation with poor prognosis. We analyzed the data of the expression of IGF2BP3 by various databases, such as TCGA and CESC microarrays (GSE7803、GSE9750 and TCGA&GETx; Fig. 1D–F), and concluded that the expression of IGF2BP3 in CC tissue is higher than normal tissue. In addition, we ascertained if the clinical features of CC patients was correlated with the expression of IGF2BP3. Patients were made a distinction between high IGF2BP3 and low IGF2BP3 groups according to the median. Statistical data demonstrated that IGF2BP3 was in positive connection with tumor lymph node, grade and stage of CC sufferers (Table 1).

**Table 1:** Relationship between IGF2BP3 expression and clinicopathological parameters in cervical cancer.

	Low IGF2BP3		High IGF2BP3		*P*-value
	No.	%	No.	%	
All patients	54	50.00	54	50.00	
Age (years)					
≤40	10	9.26	7	6.48	0.4280
>40	44	40.74	47	43.52	
Lymph node					
N0	54	50.00	48	44.44	**0.0117**
N1	0	0.00	6	5.56	
Grade					
–	12	11.11	0	0.00	**0.0069**
1	5	4.63	4	3.7	
1–2	2	1.85	1	0.93	
2	15	13.89	26	24.07	
2–3	4	0.04	3	2.78	
3	16	14.82	20	18.52	
Stage					
I	54	50.00	42	38.89	**0.0002**
II or III	0	0.00	12	11.11	

Bold values represent significant statistical differences.

### The downregulation of IGF2BP3 restrains proliferation and metastasis in CC cell lines

**Fig. 2:**
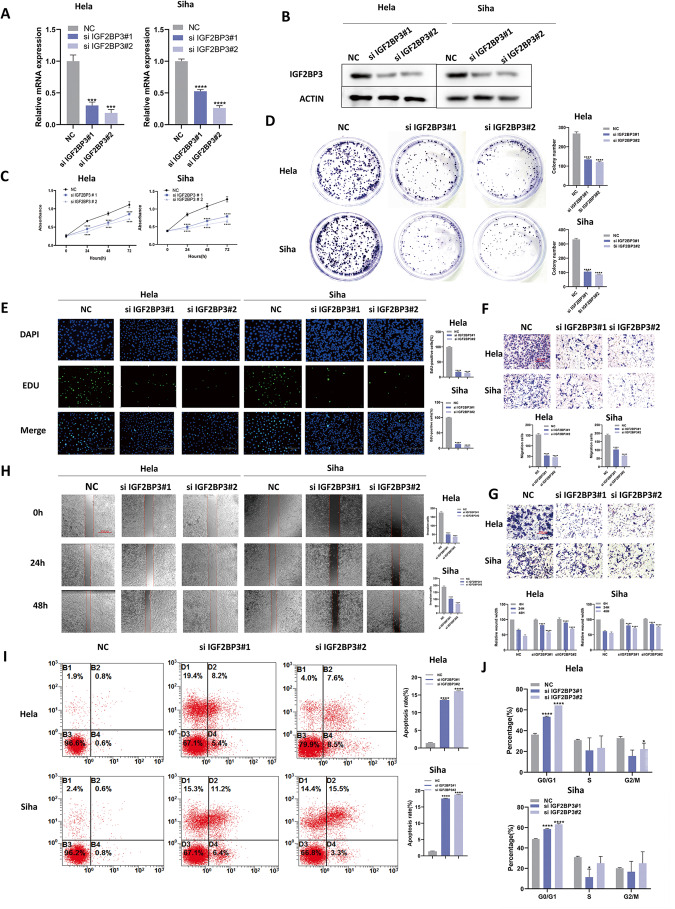
The downregulation of IGF2BP3 restrains proliferation and metastasis in CC cell lines. **A**, **B** Hela and Siha cells were transfected with IGF2BP3 siRNA, after which IGF2BP3 mRNA (**A**) and protein (**B**) levels were determined by qPCR and western blot, respectively. **C**–**E** After IGF2BP3 silencing, the viability (**C**), colony-forming ability (**D**), and proliferative ability (5-ethynyl-2′-deoxyuridine [EdU] assay) (**E**) of the cells were evaluated. Scale bars: 150 µm. **F**–**H** The effects of IGF2BP3 silencing on Hela and Siha cell migration and invasion were evaluated by transwell assays (**F**, **G**) and the wound-healing assays (**H**). Scale bars: 150 µm. (**I**, **J**) Hela and Siha cells were transfected with IGF2BP3 siRNA, then detected cell apoptosis (**I**) and appraised cell cycle by propidium iodide (PI) staining (**J**) by flow cytometry. Each value represents the mean ± SD of triplicate samples (Student’s t-test). *P < 0.05, ***P < 0.001, and ****P < 0.0001.

For the sake of seeking the effect of IGF2BP3 on CC, IGF2BP3 in Hela and Siha cells was knocked down by siRNA. We measured the transfection efficiency of IGF2BP3 siRNA using quantitative real-time reverse transcriptionquantitative PCR (qRT-PCR) qRT-PCR (Fig. 2A) and western blot (Fig. 2B). The CCK8 (Fig. 2C), colony-forming (Fig. 2D), and proliferative abilities through ethynyl-2′-deoxyuridine (EdU) assay (Fig. 2E) showed that IGF2BP3 knockdown weakened the viability of CC cells. We performed transwell assays and the wound-healing assays to demonstrate the CC cells migration and invasion were lower following IGF2BP3 knockdown (Fig. 2F–H). Flow cytometric analysis revealed that the rates of cell apoptosis (Fig. 2I) and cell cycle arrest in the G1 phase (Fig. 2J) were higher than in IGF2BP3-depleted Hela and Siha cells. Taken together, our findings suggested that IGF2BP3 knockdown weakens the proliferation and metastasis of CC cells.

### IGF2BP3 promotes lipid metabolism in CC cells

**Fig. 3:**
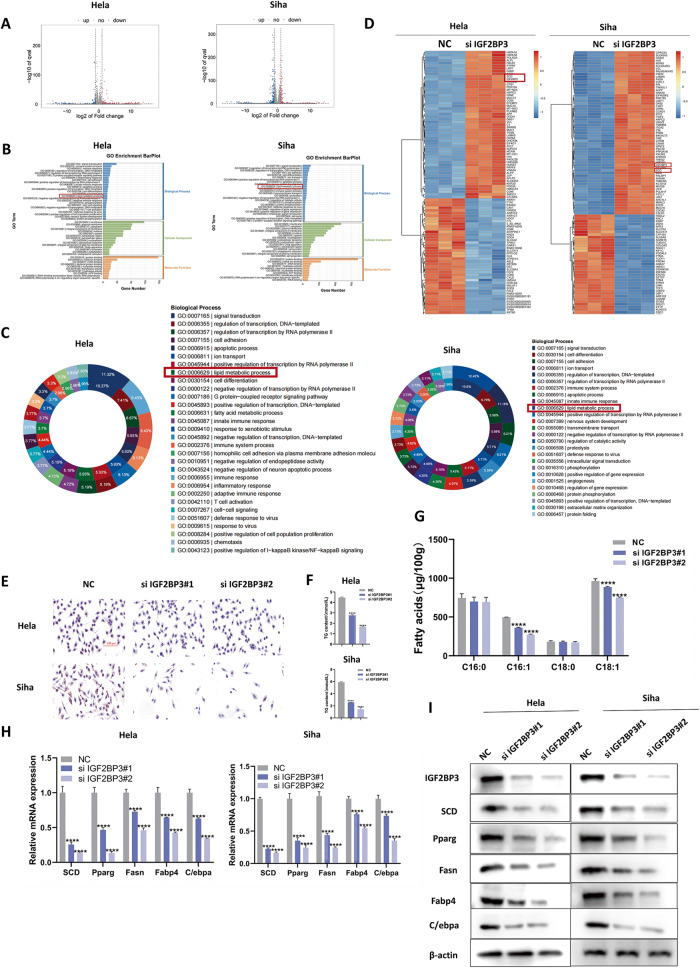
IGF2BP3 promotes lipid metabolism in CC cells. **A** Identification of the genes regulated by IGF2BP3 in CC using RNA-Seq analysis. An enhanced volcano plot was produced by the OmicStudio tools (https://www.omicstudio.cn/tool). The represent upregulated and downregulated genes are respectively displayed through red and blue dots (fold change >2 or <0.5, *p* < 0.05). **B** Gene Ontology (GO) barplot analysis of the relevant pathways in Hela and Siha cells enriched using RNA-seq. **C** A doughnut map was on the basis of Gene Ontology (GO) enriched differential genes. **D** A heat map was constructed based on the genes differentially expressed between the control and IGF2BP3-knockdown cells. **E** Oil red O staining is applied after the induction of adipogenesis. The lipid droplets are stained red and nuclei are stained blue through hematoxylin after IGF2BP3 silencing. Scale bars: 200 µm. **F**, **G** Cellular TG content (**F**) and determination of cellular fatty acids after adipogenesis by GC–MS (**G**) in CC were measured following induction of adipogenesis with IGF2BP3 knockdown. **H**, **I** qRT-PCR (**H**) and western blot (**I**) analysis of lipid metabolism marker genes and their products in Hela and Siha cells.Each value represents the mean ± SD for triplicate samples (Student’s *t* test). ****P < 0.0001.

IGF2BP3 is a member of Insulin-like growth factor 2 mRNA-binding proteins (IGF2BPs) and plays the role of RNA-binding proteins (RBPs). For the sake of exploring detailed mechanisms of IGF2BP3 driving in CC progress, we use RNA-seq in IGF2BP3-knockdown and control cells, Along with IGF2BP3 knockdown, 138 and 397 genes expression levels decreased more significantly in Hela and Siha cells than in NC cells (Fig. 3A). The results that IGF2BP3 was related to lipid metabolic process were analyzed by the barplot map, which is derived from the Term of BP, CC, MF were sorted from large to small, according to the number of differential genes annotated, then selecting the Term of Top25, Top15 and Top10 for drawing and displaying respectively (Fig. 3B). A doughnut map comed from that we drew the GO entries of each Top 20 with the largest number of up-regulated genes and down-regulated genes in the Biological Process in the GO enrichment analysis results, and proportion of GO Term genes was marked on the circle, which displayed similar conclusions above (Fig. 3C). The heat map of the differential genes expression (Fig. 3D) reveal SCD serves as a downstream target of IGF2BP3. Furthermore, depletion of IGF2BP3 causes cells had markedly less lipid droplets (Fig. 3E), and cellular triglyceride (TG) contents decreased accordingly (Fig. 3F). In order to investigate whether IGF2BP3 could change aerobic the fatty acids during lipid metabolism in CC, the contents of palmitoleic acid (C16:1) and oleic acid (C18:1) (Fig. 3G) were decreased following IGF2BP3 knockdown. In addition, the mRNA and protein levels of lipid metabolism marker genes (Pparg, Fasn, Fabp4, and C/ebpα), proved respectively by qRT-PCR and western blot decreased significantly in CC cells (Fig. 3H, I).

### IGF2BP3 stimulates SCD expression by recognizing the m6A-modified site in SCD RNA in CC cells

**Fig. 4:**
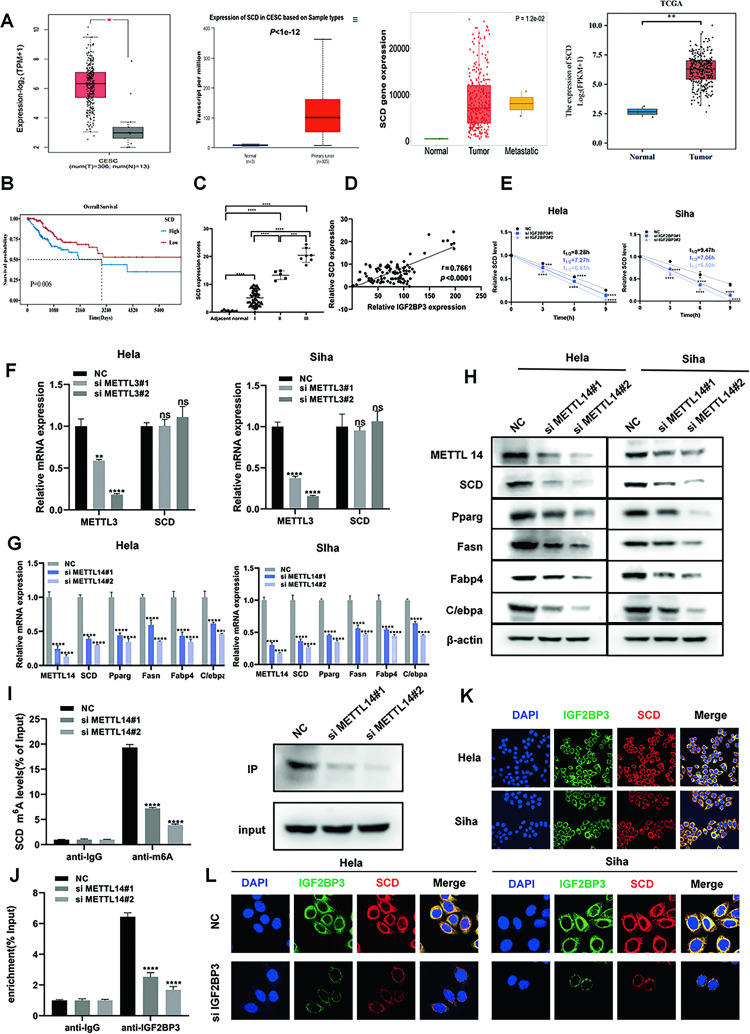
IGF2BP3 stimulates SCD expression by recognizing the m6A modified site in SCD RNA in CC cells. **A** Differences in SCD expression between CC tissue and normal tissue were analyzed through the GEPIA ((http://gepia.cancer-pku.cn/detail.php)), UALCAN (http://ualcan.path.uab.edu/), and TNMplot (https://tnmplot.com/analysis/). **B** Survival curve presents the overall survival probability of high SCD and low SCD using TCGA databases. **C** The expression of SCD in different stages of CC and adjacent normal tissues (*n* = 108) (Student’s *t* test). **D** Correlation between IGF2BP3 and SCD expression in CC in tissue microarrays. **E** The attenuation rates of SCD mRNA and qPCR at the indicated point in time after Hela and Siha cells were treated with actinomycin D (5 g/ml) when IGF2BP3 was restrained. **F** qRT-PCR tested the METTL3 and SCD relative mRNA expression. **G**, **H** The relative mRNA and protein expression of METTL14, SCD, and adipogenesis-related genes were determined using qRT-PCR (**G**) and western blot (**H**). **I** m6A-RIP assay and IP assays were performed to detect the SCD m6A levels in Hela cells after METTL14 silencing. **J** The binding of IGF2BP3 to SCD was verified through the RIP assay. **K**, **L** Expression of IGF2BP3 (green) and SCD(red) in control (**K**) and IGF2BP3-knockdown (**L**) cells according to confocal microscopy. Each value represents the mean ± SD for triplicate samples (Student’s *t* test). *P < 0.05, **P < 0.01, ***P < 0.001, ****P < 0.0001, and ns means no significance.

The GEPIA, University of ALabama at Birmingham Cancer data analysis Portal (UALCAN), TNMplot, and TCGA databases analyzed that the expression of SCD in CC tissue is higher than that in normal tissue (Fig. 4A) and directly related to bad prognosis (Fig. 4B). Then IHC staining of SCD in 108 patients tissues comparing with adjacent normal tissues showed that SCD expression was obviously higher in CC tissues (Fig. 4C and Table 2). The scatter plot demonstrates that IGF2BP3 has the positively correlation with SCD (Fig. 4D). The regulatory effect of IGF2BP3 on the stability of SCD mRNA was detected by RNA stability analysis. Just as expected, the knockdown of IGF2BP3 decreased the half-value period of SCD mRNA (Fig. 4E). Adenosine was targeted by several nucleic acid-decorated metals in order to form methyladenosine [12, 13]. Probably the most typical of these are METTL3 and METTL14, and these compounds form aheterocomplex with WTAP (Wilms’Tumor1-AssociatingProtein), then m6A is formed in mRNA [13–25]. The expression of SCD in CC cells did not decrease after METTL3 knockdown (Fig. 4F). METTL14 is a noncatalytic subunit of the N6-adenosine-methyltransferase complex which can regulate mRNA function and stabilize mRNA transcripts to accelerate tumor progression. The expression products of lipid-metabolism-related factors were strikingly decreased after METTL14 knocked down in CC cells, especially SCD (Fig. 4G, H). What is more, the m6A-RIP assay was performed in METTL14-knockdown Hela cells for the sake of exploring if reducing m6A levels can affected SCD expression. qRT-PCR and IP assays reflected that the levels of m6A-modified SCD were significantly downregulated after METTL14 knockdown (Fig. 4I). The RIP results further reflected that IGF2BP3-bound SCD mRNA expression was decreased in METTL14-silenced Hela cells (Fig. 4J). Expression of IGF2BP3 and SCD in Hela and Siha cells were demonstrated by Confocal microscopy (Fig. 4K). Moreover, the expression decreased in IGF2BP3-knockdown cells compared with that in the NC group (Fig. 4L). Above all, the results conclude that IGF2BP3 regulates SCD mRNA methylation via IGF2BP3-METTL14 complex then regulating lipid metabolism.

**Table 2:** Relationship between SCD expression and clinicopathological parameters in cervical cancer.

	Low SCD		High SCD		*P*-value
	No.	%	No.	%	
All patients	54	50.00	54	50.00	–
Age (years)					
≤40	10	9.26	7	6.48	0.4280
>40	44	40.74	47	43.52	
Lymph node					
N0	54	50.00	48	44.44	**0.0117**
N1	0	0.00	6	5.56	
Grade					
–	11	10.19	1	0.93	**0.0013**
1	7	6.48	2	1.85	
1–2	1	0.93	2	1.85	
2	20	18.52	21	19.44	
2–3	5	0.05	2	1.85	
3	10	9.26	26	24.07	
Stage					
I	54	50.00	42	38.89	**0.0002**
II or III	0	0.00	12	11.11	

Bold values represent significant statistical differences.

### SCD overexpression partially rescued IGF2BP3 knockdown-caused proliferation, metastasis, and lipid metabolism in CC cells

**Fig. 5:**
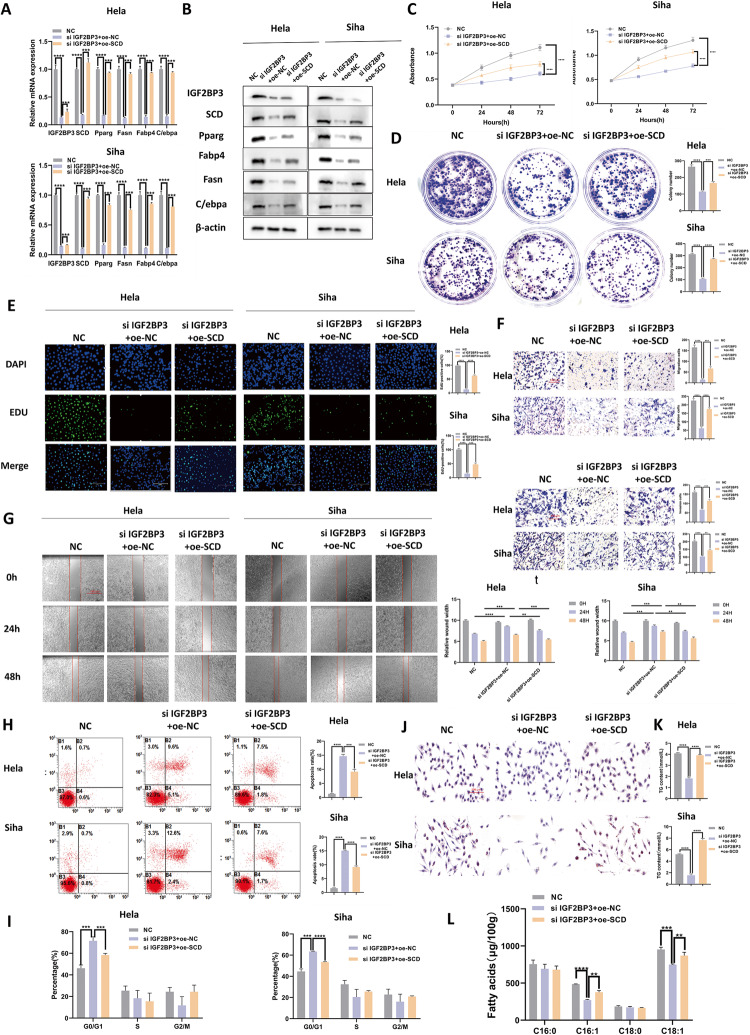
SCD overexpression partially rescued the IGF2BP3 knockdown-mediated drop in CC cell proliferation. **A** The mRNA expression levels of SCD and adipogenesis-related genes were evaluated by qRT-PCR with transfecting SCD expression plasmids. **B** Western blot-based evaluation of the protein levels of SCD and adipogenesis-related genes in Hela and Siha cells expressing siIGF2BP3 after transfecting SCD expression plasmidsa. **C**–**E** Cell viability,clonogenicity and proliferation of siIGF2BP3-expressing Hela and Siha cells transfected or not with oe-SCD as monitored by CCK-8 assay (**C**), colony formation assay (**D**), and EdU staining assay respectively (**E**). **F**, **G** Migration and invasion assays of siIGF2BP3-expressing Hela and Siha cells transfected or not with oe-SCD as assessed using transwell assays (**F**) and the wound-healing assays (**G**). **H**, **I** Flow cytometry was used to detect cell apoptosis (**H**) and cell cycle by propidium iodide (PI) staining (**I**) in siIGF2BP3-expressing Hela and Siha cells plasmid-transfected or not with oe-SCD. **J**–**L** SCD was overexpressed in CC cells transfected with siIGF2BP3, then the lipid droplets (**J**), cellular TG content (**K**) and the cellular fatty acids (**L**) were tested. Each value represents the mean ± SD for triplicate samples (Student’s *t* test). **P < 0.01, ***P < 0.001, and ****P < 0.0001.

It is quite possible that SCD is a downstream target of IGF2BP3, so IGF2BP3 knockdown CC cells were transfected with SCD-over-expressing plasmids. First, we discovered by qRT-PCR (Fig. 5A) and western blot (Fig. 5B) that the decrease in the expression of SCD and lipid-metabolism-related genes was recovered. Further, the losses of CC cell viability (Fig. 5C) and clonogenicity (Fig. 4D) resulting from the knockdown of IGF2BP3 in vitro were rescued by SCD overexpression. Simultaneously, overexpressing SCD rescued the IGF2BP3-knockdown-caused reduction in the proliferation in CC cells by the EdU assay (Fig. 4E). Then SCD overexpression partially rescued the IGF2BP3 knockdown-mediated drop in the migratory and invasive ability of CC cells using transwell assays (Fig. 5F) and the wound-healing assays (Fig. 5G). The cell apoptosis (Fig. 5H) and cell cycle (Fig. 5I) were detected by flow cytometry, which obtained similar results. In order to study the influence of SCD on lipid metabolism disorder in CC cells, lipid metabolism indicators were tested. Unsurprisingly, SCD overexpression rescued the lipid droplets (Fig. 5J), TG contents (Fig. 5K), and the contents of palmitoleic acid (C16:1) and oleic acid (C18:1) (Fig. 5L) with the inhibitory effect on lipid metabolism compared with the IGF2BP3 knockdown cells. In conclusion, these results showed that the losses of proliferation, metastasis, and lipid metabolism in the IGF2BP3 knockdown cells are partially restored by SCD to a certain degree.

### IGF2BP3 enhances CC tumor growth in vivo

**Fig. 6:**
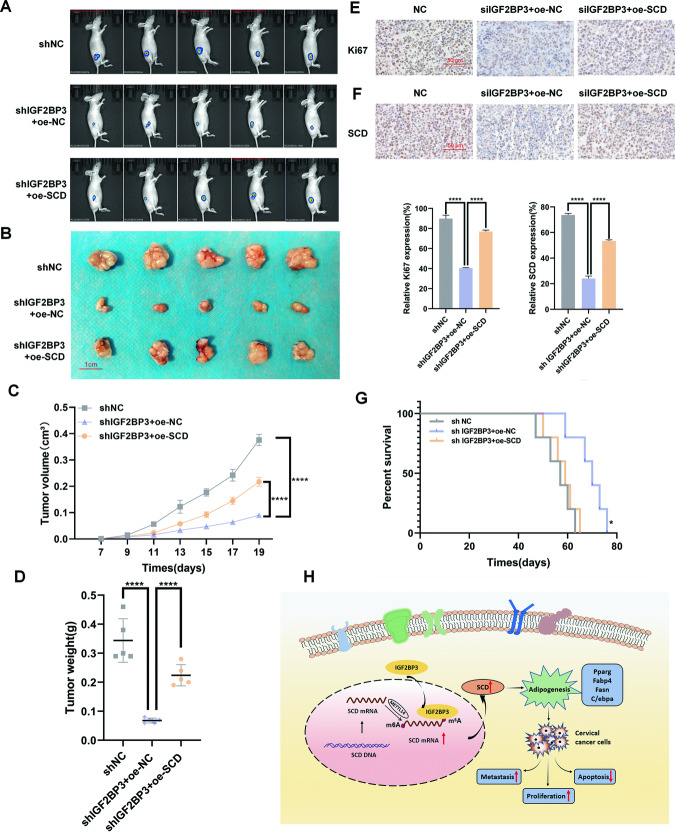
IGF2BP3 enhances the growth of CC tumor in vivo. **A**, **B** Hela cells stably expressing shNC, shIGF2BP3, or shIGF2BP3+oe-SCD were subcutaneously injected into the right back of nude mice. Tumor images were captured using the PerkinElmer IVIS preclinical in vivo imaging system 20 days (**A**) and 30 days (**B**) after injection. **C** The tumor volumes were measured every two days from the 7th day after injection. **D** The weights of transplanted tumors were measured. **E** The tumor sections were stained by IHC with an antibody against Ki-67. Scale bar: 50 μm. **F** The tumor sections were stained by IHC with an antibody against SCD. Scale bar: 50 μm. **G** Kaplan–Meier survival curve presents the overall survival rate of mice in each group (*n* = 5, *P < 0.05 through the log-rank test). **H** A proposed modulatory mould for the function of IGF2BP3 in adipogenesis and tumorigenesis. Each value represents the mean ± SD (Student’s *t* test). *P < 0.05 and ****P < 0.0001.

We built a mouse model to investigate the role of IGF2BP3 in vivo. Hela cells expressing shNC, shIGF2BP3+oe-NC, shIGF2BP3+oe-SCD were subcutaneously injected into 5-week-old nude mice. In all, 7 days after injection, the mice tumor size were observed daily. We measured tumor volumes every 2 days and plotted a growth curve. And tumors were imaged in vivo on the 25th day (Fig. 6A). We saw that the injection of Hela cells expressing shIGF2BP3+oe-NC reduced significantly the tumor size than those of shNC cells, however mice injected with shIGF2BP3+oe-SCD-expressing hela cells had slightly increased tumors (Fig. 6B). We got similar results when the nude mice were executed and tumors were resected 30 days after injection (Fig. 6C). As the Fig. 6D presented, shIGF2BP3+oe-NC reduced tumor weight, whereas shIGF2BP3+oe-SCD increased tumor weight. Then, the shows of Ki-67 IHC staining, applied to estimate tumor proliferation, were in accordance with the aforementioned discoveries (Fig. 6E). Next, IHC staining of mouse tumor tissue using the SCD antibody showed that SCD expression diminished following IGF2BP3 knockdown (Fig. 6F). Furthermore, Kaplan–Meier survival curve analysis revealed that the survival time of the shIGF2BP3+oe-NC group was increased, but the injection of Hela cells expressing shIGF2BP3+oe-SCD group and the shNC resulted in the nude mice survival time to a similar degree (Fig. 6G). We also established a nude mouse tumor-bearing model with another cell line Siha, and the results were shown in the Supplementary Figure (Fig. S). We come to a conclusion that the proposed modulatory model of IGF2BP3 in CC, in which IGF2BP3 regulates the methylation of SCD mRNA by IGF2BP3-METTL14 complex, thereby accelerating lipid metabolism and tumor progression in CC (Fig. 6H).

## Discussion

Cervical cancer is the most common gynecological malignant tumor, which is concentrated in countries with low and medium development level and has a high mortality rate. The number of new cervical cancer cases in China accounts for ~1/3 of the global total each year. In recent years, the trend of rejuvenation has obviously increased, with an annual growth rate of 2–3%, which seriously threatens the health of Chinese women. Thus, the search for key moleculesis is of great significance to further clarify the pathogenesis of cervical cancer and provide new strategies for the prevention and treatment of cervical cancer. During this research, the potential effect of IGF2BP3 in CC progression were defined in vitro as well as in vivo, at the same time, the function of IGF2BP3 in lipid metabolism was explored unprecedentedly. The results revealed probable treatment strategies referring to the targeting of metabolic pathways.

IGF2BP3 is a member of IGF2BPs family, and plays the role of RNA binding protein (RBPs). RNA-binding proteins are one of the ways of post-transcriptional regulation. By recognizing the interaction between special RNA-binding domains and RNA, they are widely involved in the regulation processes of RNA shearing, transport, sequence editing, intracellular localization, and translation control, and play an important regulatory role in cell physiology and pathology. Evidence shows that RBPs are involved in the regulation of most cancer markers, promoting cell proliferation, cell death resistance, tumor stem cell dryness maintenance, cell migration and immune escape [16]. Besides, the main function of IGF2BPs is to bind to m6A site of RNA, maintain the stability of mRNA and improve the translation level of target mRNA [17]. And it has been reported that IGF2BP3 can promote cervical cancer cell proliferation by binding to the mRNA of RAB2B [18]. Additionally, IGF2BP3 stimulates tumorigenesis by RCC2 in acute myeloid leukemia [19]. The role of IGF2BP3 modulates laryngeal squamous cell carcinoma progression through TMBIM6 mRNA [20]. Moreover,Tumor immune monitoring is inhibited by IGF2BP3 through targeting breast cancer PD-L1 mRNA [21]. It is IGF2BP3 that targets cyclin D1 and VEGF to regulate the cell cycle and angiogenesis of colon cancer [22]. Also, FGF13-AS1 inhibits stem cell capacity and glycolysis in breast cancer cells by IGF2BPs [23]. Nevertheless, its function in lipid metabolism is still indistinct. Here in this study, we analyzed the performance of IGF2BP3 both in vitro and in vivo. It is the first time our results prove that IGF2BP3 upregulates the proliferative, metastatic, and adipogenetic capacity of CC cells through regulating SCD.

Fat metabolism is a crucial part of tumor energy metabolism. It has been shown that fat metabolism is relevant to higher life quality of patients with cancers and longer survival expectation [24]. Studies in gastric carcinoma have revealed that triggered off cancer-relevant cachexia are driven by suppressed lipid metabolism and increased brown adipose differentiation by exosomal miR-155 with C/EPBβ [25]. Ding et al. reported that circPTK2, in cancer-associated cachexia, stimulated lipolysis and decreased lipid metabolism by a ceRNA mechanism [26]. Fatty acid metabolism and its related lipid metabolic pathways are closely related to the malignant progression of CC. In particular, it involves the synthesis, uptake, activation, oxidation, and transport of fatty acids [27]. FABP5 (fatty acid-binding protein 5) was markedly upregulated in CC with LNM and correlated with poor prognosis. Furthermore, FABP5 promoted epithelial-mesenchymal transition, lymphangiogenesis, and LNM by reprogramming fatty acid (FA) metabolism [28]. LNMICC (lncRNA associated with lymph node metastasis in cervical cancer) which promoted lymph node metastasis by reprogramming fatty acid metabolism acted as a candidate prognostic biomarker and therapeutic target in cervical cancer [29]. Fatty acid synthase expression is related to tumor-infiltrating immune cells in tumors (e.g., CD8 + T-cell infiltration level in cervical squamous cell carcinoma) [30]. High levels of DECR1 (2,4-Dienoyl-CoA reductase) serves to enhance lipolysis and the release of fatty acid energy stores to support cervical cancer cell growth [31]. As SCD is a essential regulator of lipid metabolism [32], the influence of IGF2BP3 on lipid metabolism in CC was assessed. Coming after IGF2BP3 knockdown,we found that proliferation, metastasis, and lipid metabolism in CC cell decreased. Simultaneously, the restrainment of lipid metabolism and CC process was rescued following SCD-overexpression, indicating that SCD acts as a bridge between IGF2BP3 and adipogenetic modulation. In view of the fact that cell metabolism is a complicated process including a great deal of genes movements, it cannot be ruled out that IGF2BP3 maybe aim at lipid metabolism through affecting mRNAs except SCD methylation state.

To sum it up, we displayed that IGF2BP3 expression was obviously higher in CC tissues and directly related to CC stage using tissue microarray (TMA) staining. Moreover, RNA-seq demonstrated that the lipid metabolism-relevant pathways in IGF2BP3 knockdown Hela and Siha cells obviously enriched. Further, the downregulation of IGF2BP3 restrains proliferation, metastasis, and lipid metabolism in CC both intracorporeal and extracorporeal tests. Mechanistically, IGF2BP3 influences lipid metabolism by combining with SCD through N6- methyladenosine (m6A) modification. Besides, it was proved that the proliferation, metastasis, and lipid metabolism ability of CC cells with IGF2BP3 knockdown could be partially remedied by SCD overexpression. All in all, the studying which the IGF2BP3/SCD/lipid metabolism axis maybe crucial to the generation mechanism of CC reveals that an innovative and effective remedial method used for treating CC patients targets IGF2BP3 and SCD.

## Materials and methods

### Cell culture

The human CC cell lines HeLa and SiHa were cultured in Dulbecco’s modified Eagle’s medium (DMEM) (Gibco, USA) supplemented with 10% FBS (Gibco) at 37 °C under 5% CO2. Both cell lines were tested to be mycoplasma-negative and were authenticated using short tandem repeat (STR) profiling [33].

### RNA interference and lentivirus infection

We bought small interfering RNAs (siRNAs) targeting IGF2BP3, SCD, and methyltransferase-like 14 (METTL14) from GenePharma (Shanghai, China). SiRNA and negative control (NC) siRNA was transfected with Lipofectamine 3000 (Invitrogen, USA) ~48 h. qRT-PCR and western blotting were used to verify the transfection efficiency.

Next, the pHBLV-U6-MCS-EF1-Luc-T2A-Puro vector (HANBIO, Shanghai, China) inserted IGF2BP3-shRNA lentivirus, then the lentiviruses-infected CC cells grew in DMEM containing 5 µg/ml polybrene then. IGF2BP3-knockdown cells were transfected with the SCD-overexpression plasmid to rescue. Stable cell lines were produced by selection using 10 µg/ml puromycin (Solarbio, China). The aforesaid sequences appear in Table S1.

### Quantitative real-time reverse transcription quantitative PCR and RNA-seq analysis

We isolated total RNA from prepared CC cells by an RNA isolation kit (TSINKGE, Beijing, China), then reversed transcription as cDNA using one-step RT-PCR. The SYBR Green PCR Master Mix (Vazyme Biotech, Nanjing, China) was applied when Real-time qPCR was at execution time. The used primer sequences are listed in Table S2. IGF2BP3 siRNA-transfected Hela cells were collected and sent to Lc-Bio Technologies (Hangzhou) Co, Ltd (China) for sequencing to screen significant genes.

### Western blot and immunoprecipitation assays

Western blot measured the expression of protein as mentioned earlier [34]. Following blocking, the polyvinylidene difluoride membranes were incubated with different primary antibodies (Table S3) spent the night at 4 °C. The original picture can be found in western.zip.

For co-IP, we used a lysis buffer including protease and phosphatase inhibitor to lyse transfected hela cells. Then we collected the supernatant following centrifugation of the lysed cells at 4 °C for 10 min. Adding the anti-m6A and IgG antibodies to the lysate with Protein A/G PLUS-Agarose, we incubated them overnight. Lastly, the beads were gathered and implemented by western blot.

### Cell viability and colony formation assays

Cell Counting Kit-8 (CCK-8) assay (Yisheng, China) was used to analyze cell viability. Eight thousand transfected cells were inoculated into each well of 96-well plates. Then we incubated 2 h with DMEM containing 10% CCK-8 reagent before measuring the absorbance at 450 nm.

As the colony formation assay, four hundred transfected cells were inoculated into each 60 mm Petri dish. After 2 weeks of cultivation, the generated cell colony were immobilized in 4% paraformaldehyde (PFA) and then dyed with 1% crystal violet (Solarbio) ~15 min. ImageJ software was used to calculate the cell colonies after washed with PBS [35].

### Ethynyl-2′-deoxyuridine incorporation assay

EdU assays was monitored by a BeyoClick EdU Cell Proliferation Kit with DAB (Beyotime, China). The processed cells were incubation ~2 h with 10 µM EdU, immobilized in 4% PFA ~15 min, infiltrated in PBS including 0.3% Triton X-100, and then dyed with Click Additive Solution at 37 °C for 1 h. Cell nucleus were colored by DAPI (Beyotime). Pictures were caught through an EVOS M5000 Fluorescence Microscope (Thermo Fisher Scientific, USA) [36].

### Scratch wound-healing assay

Cells transfected with siRNAs or relevant NCs were cultivated into six-well plates. As the cell fusion reached 90%, we drew a direct line wounding the cell layers with the tip of a sterile pipette, and washed using PBS a few times to get rid of cellular fragments, then went on incubating together with blood serum-free medium ~48 h. After the wounding, taking images consecutively at the scratch identical position was at 0, 24, and 48 h. The rate of wound healing = [(the wound width of 0 h–48 h)/0 h wound width] × 100% [37].

### Transwell migration and invasion experiments

The transwell migration and invasion assay was measured in transwell chambers (Thermo Fisher Scientific). Transfected SiHa and HeLa cells were conducted with mitomycin C, seeded in the upper chamber in 100 μL of blood serum-free medium. A complete medium (DMEM containing 10% FBS) was placed into the lower chamber as a chemoattractant simultaneously. After incubated for 24 h at 37 °C, the culture medium was removed, then the cells that passed through the filter were fixed with 4% paraformaldehyde, stained with 1% crystal violet, and photographed by an EVOS M5000 Fluorescence Microscope.

The transwell invasion assay was proceeded according to the foregoing stated, with the exception of 100 μL of 1:8 DMEM-diluted Matrigel (BD, USA) added to each well before seeding the cells on the membrane and then incubation for 48 h.

### RNA immunoprecipitation assay

It was RNA immunoprecipitation (RIP) [38] that was monitored by a RIP-Assay Kit (MBL Life Science, Japan). Transfected hela cells were seeded into a 15 cm culture dish ~48 h, before gathered by cell scrapers and suspension with nuclease-free PBS. As co-immunoprecipitation, we shifted the precleared cell lysate to the EP tube including antibodies (IGF2BP3 or IgG) immobilized Protein A/G PLUS-Agarose beads, and washing by lysis buffer once, then the incubation tube was rotated at 4 °C for 3 h. After centrifugation, the antibody-immobilized beads-RNP complex was washed using a wash buffer. RNA after isolation and purify was performed using qRT-PCR.

### m6A-RNA immunoprecipitation assay

For m6A-RNA immunoprecipitation, we pulled down m6A-modified SCD using an N6-methyladenosine (m6A) antibody (Active Motif, China). A polyA Spin mRNA Isolation Kit (NEB, China) purified total RNA extractive from Hela cells after treatment. Protein A/G PLUS-Agarose beads were incubated (m6A antibody and IgG) by IP buffer as described above [39]. Then the depurated RNA was put in the aforesaid EP tube including proteinase and RNase inhibitor, then hatched at 4 °C through a night. We made qRT-PCR analysis after RNA extracted by an RNA extraction kit, using the primers presented in Table S2. The IP assay embodied the protein expression of m6A. In short, we added 100 µl of the antibody-immobilized beads-RNP complex to a fresh EP tube, tackled using SDS–PAGE, and performed western blot.

### Immunohistochemistry

We stained the CC tissue microarrays (TMAs) with antibodies targeting IGF2BP3 (1:50, Proteintech), SCD (1:100, Proteintech), and Ki-67 (1:300; Abcam) with the early descriptive approach [40]. Dyed sections were scanned by 3DHISTECH imaging system (Hungary) and analyzed by Indica Labs software (USA). Measuring areas of tissue slices were read voluntarily using the Servicebio image analysis system, and the histochemistry score (H-score) was calculated. The formula is as follows: H-score = Σ(pi×i)= (percentage of weak intensity × 1) + (percentage of moderate intensity × 2) + (percentage of strong intensity × 3). Aipathwell was used as the analysis software.

### Flow cytometry

We examine the influences of IGF2BP3 and SCD on apoptosis (through an Annexin V-FITC/PI apoptosis double staining kit), and cell cycle, analyzed by PI/RNase staining buffer (BD, USA), of treated properly HeLa and SiHa cells by Flow cytometry. Acquisition and analysis of data were carried out by NovoExpress software (ACEA Biosciences, USA).

### Oil Red O staining

The treated cells were immobilized in 4% paraformaldehyde (PFA) for 30 min after lipid metabolism. We wash cells using 60% isopropanol for 30 s after PBS twice. Next, the oil red O reagent stained cells ~15 min to observe the lipid droplets at room temperature. Hematoxylin solution counterstained cells afterwards and we captured images using a microscope ultimately.

### Measurement of TG

TG content was monitored using Triglyceride (TG) assay kit (Nanjing Jiancheng Bioengineering Institute, A110-1–1). The cells were washed with isotonic buffer, and then centrifuged for cell precipitation. A buffer containing 1% Triton X-100 in PBS lysed the cells. We added TG assay reagent to the orifice plate and incubated for 10 min. The absorbance of each hole was measured by enzyme-labeled instrument at 500 nm and the TG content was calculated following the manufacturers’ protocols [41].

### Measurement of fatty acids

The total lipids extraction was performed by using mixed solution of ether and petroleum ether. Fatty acid methyl esters (FAMEs) were produced by extractive lipins by a way on the basis of 14% boron trifluoride-methanol solution [42]. We measure fatty acids using Q Exactive™ GC Orbitrap™ GC–MS/MS (Thermo Scientifc) with the TG-FAME (50 m × 0.25 mm × 0.20 μm) capillary column. Sample inlet temperature was 260 °C, the temperature of oven is set to heat up from 80 °C (holding for 1 min) to 160 °C at a rate of 20 °C /min, and then to 230 °C (6 min) at a rate of 5 °C/min. Compared with known standards (Sigma Chemical), the fatty acids peaks were distinguished. Finally, fatty acids were quantitatively described.

### RNA stability assay

In 12-well plates cultured transfected CC cells. After the cells adhered, 5 μg/mL actinomycin was used to cope with the cells for 0, 3, 6, and 9 h. After that, we isolated total RNA from prepared CC cells by an RNA isolation kit (TSINKGE, Beijing, China). qRT-PCR was utilized to ascertain the difference of mRNA expression level and half-life length at specific different time points.

### Immunofluorescence assays

When the cell density is 30%, the cells are inoculated into a 15 mm glass-bottomed cell culture dish. Before fixing the cells by 4% formaldehyde, they were washed through phosphate-buffered saline (PBS) three times. Next, it is 0.2% Triton X-100 that we permeabilized ~10 min the treated cells with. In all, 4% BSA was blocked at 37° for 30 min, and PBS was used to wash the cells three times. Under the condition of 4°, we incubated the cells using anti-SCD rabbit polyclonal antibody overnight. PBS washed the cells three times. At room temperature, the cells were incubated with mixed anti-BAP31 mouse monoclonal antibody labeled with Cy5 and donkey anti-rabbit IgG labeled with Cy3 for 1.5 h. Under room temperature conditions, we incubated the cells using mixed Cy5 labeled anti-IGF2BP3 mouse monoclonal antibody and Cy3 labeled donkey anti-rabbit IgG ~2 h. After PBS rinsed the cells three times, nuclear staining was performed with 4′,6-diamidino-2-phenylindole (DAPI). Finally, an FV-1000/ES confocal microscope (Olympus, Tokyo, Japan) was employed to analyze our all samples.

### Animal experiments

We randomly divided Female BALB/c nude mice (4–6 weeks old) into 3 groups (*n* = 5 per group) via block pseudo-randomization to build a xenotransplantation oncogenesis model. 5 × 10^6^ CC cells had stable expressions of shNC, shIGF2BP3+oe-NC, and shIGF2BP3+oe-SCD, then subcutaneous injection to every nude mouse right back. Following tumor neoplasia, we took a measurement of tumor volume one day apart using V = 1/2 × length × width^2^. The investigators were blinded to grouping assignment. A PerkinElmer IVIS preclinical in vivo imaging system analyzed images. The protocol for the animal experiments complied with the ARRIVE guidelines and was examined for approval by The Ethics Committee of the Fourth Military Medical University.

### Statistical analysis

Sample size calculation was not carried out, but the sample size is based on the similar research of heterogeneous transplantation mode analysis used before [43, 44]. As statistical analysis, We used GraphPad Prism 8.0.2. To contrast two groups, Students’ *t* test was carried out. We used one-way ANOVA to compare numerous groups. The variance was similar between the groups and was statistically compared. Kaplan-Meier method was performed to count the survival probability. Every experiment was repeated three times at least and the data were presented as means ± SD. *P < 0.05, **P < 0.01, ***P < 0.001, and ****P < 0.0001 were considered statistically significant. NS indicated not significant.

### Supplementary information

Supplemental Material

Supplemental Material-western

Reproducibility checklist

## Data Availability

All the data generated or analyzed in this study are contained in this published article [and its supplementary information files].
