# Supplementary material for: IGF2BP3 enhances lipid metabolism in cervical cancer by upregulating the expression of SCD
Source: Cell Death Dis. 2024 Feb 14;15(2):138. doi: 10.1038/s41419-024-06520-0 (PMC10867090; doi:10.1038/s41419-024-06520-0)
Supplement: Supplementary file 1 — Supplemental Material [file 41419_2024_6520_MOESM1_ESM.docx]

***Supplementary Table 1. The sequences of siRNAs, shRNAs and plasmids***

| Plasmids | sequences (5' > 3') |
| --- | --- |
| siIGF2BP3#1  si/shIGF2BP3#2  siMETTL3#1  siMETTL3#2  siMETTL14#1  siMETTL14#2  siNC/NC  pHBLV-U6-MCS-EF1-Luc-T2A-Puro/SCD | UUCCUGCAAUGGAGAUAUCTT  CUUUGUUAGUCCUAAAGAATT  GCACTTGGATCTACGGAAT  CGACTACAGTAGCTGCCTT  GCTGGACTTGGGATGATATTA  GAACCTGAAATTGGCAATATA  UUCUCCGAACGUGUCACGUTT  ATGGCCGATGCTAAGAACATTAAGAAGGGCCCTGCTCCCTTCTACCCTCTGGAGGATGGCACCGCTGGCGAGCAGCTGCACAAGGCCATGAAGAGGTATGCCCTGGTGCCTGGCACCATTGCCTTCACCGATGCCCACATTGAGGTGGACATCACCTATGCCGAGTACTTCGAGATGTCTGTGCGCCTGGCCGAGGCCATGAAGAGGTACGGCCTGAACACCAACCACCGCATCGTGGTGTGCTCTGAGAACTCTCTGCAGTTCTTCATGCCAGTGCTGGGCGCCCTGTTCATCGGAGTGGCCGTGGCCCCTGCTAACGACATTTACAACGAGCGCGAGCTGCTGAACAGCATGGGCATTTCTCAGCCTACCGTGGTGTTCGTGTCTAAGAAGGGCCTGCAGAAGATCCTGAACGTGCAGAAGAAGCTGCCTATCATCCAGAAGATCATCATCATGGACTCTAAGACCGACTACCAGGGCTTCCAGAGCATGTACACATTCGTGACATCTCATCTGCCTCCTGGCTTCAACGAGTACGACTTCGTGCCAGAGTCTTTCGACAGGGACAAAACCATTGCCCTGATCATGAACAGCTCTGGGTCTACCGGCCTGCCTAAGGGCGTGGCCCTGCCTCATCGCACCGCCTGTGTGCGCTTCTCTCACGCCCGCGACCCTATTTTCGGCAACCAGATCATCCCCGACACCGCTATTCTGAGCGTGGTGCCATTCCACCACGGCTTCGGCATGTTCACCACCCTGGGCTACCTGATTTGCGGCTTTCGGGTGGTGCTGATGTACCGCTTCGAGGAGGAGCTGTTCCTGCGCAGCCTGCAAGACTACAAAATTCAGTCTGCCCTGCTGGTGCCAACCCTGTTCAGCTTCTTCGCTAAGAGCACCCTGATCGACAAGTACGACCTGTCTAACCTGCACGAGATTGCCTCTGGCGGCGCCCCACTGTCTAAGGAGGTGGGCGAAGCCGTGGCCAAGCGCTTTCATCTGCCAGGCATCCGCCAGGGCTACGGCCTGACCGAGACAACCAGCGCCATTCTGATTACCCCAGAGGGCGACGACAAGCCTGGCGCCGTGGGCAAGGTGGTGCCATTCTTCGAGGCCAAGGTGGTGGACCTGGACACCGGCAAGACCCTGGGAGTGAACCAGCGCGGCGAGCTGTGTGTGCGCGGCCCTATGATTATGTCCGGCTACGTGAATAACCCTGAGGCCACAAACGCCCTGATCGACAAGGACGGCTGGCTGCACTCTGGCGACATTGCCTACTGGGACGAGGACGAGCACTTCTTCATCGTGGACCGCCTGAAGTCTCTGATCAAGTACAAGGGCTACCAGGTGGCCCCAGCCGAGCTGGAGTCTATCCTGCTGCAGCACCCTAACATTTTCGACGCCGGAGTGGCCGGCCTGCCCGACGACGATGCCGGCGAGCTGCCTGCCGCCGTCGTCGTGCTGGAACACGGCAAGACCATGACCGAGAAGGAGATCGTGGACTATGTGGCCAGCCAGGTGACAACCGCCAAGAAGCTGCGCGGCGGAGTGGTGTTCGTGGACGAGGTGCCCAAGGGCCTGACCGGCAAGCTGGACGCCCGCAAGATCCGCGAGATCCTGATCAAGGCTAAGAAAGGCGGCAAGATCGCCGTGTAATAA  ATGACCGAGTACAAGCCCACGGTGCGCCTCGCCACCCGCGACGACGTCCCCAGGGCCGTACGCACCCTCGCCGCCGCGTTCGCCGACTACCCCGCCACGCGCCACACCGTCGATCCGGACCGCCACATCGAGCGGGTCACCGAGCTGCAAGAACTCTTCCTCACGCGCGTCGGGCTCGACATCGGCAAGGTGTGGGTCGCGGACGACGGCGCCGCGGTGGCGGTCTGGACCACGCCGGAGAGCGTCGAAGCGGGGGCGGTGTTCGCCGAGATCGGCCCGCGCATGGCCGAGTTGAGCGGTTCCCGGCTGGCCGCGCAGCAACAGATGGAAGGCCTCCTGGCGCCGCACCGGCCCAAGGAGCCCGCGTGGTTCCTGGCCACCGTCGGCGTCTCGCCCGACCACCAGGGCAAGGGTCTGGGCAGCGCCGTCGTGCTCCCCGGAGTGGAGGCGGCCGAGCGCGCCGGGGTGCCCGCCTTCCTGGAGACCTCCGCGCCCCGCAACCTCCCCTTCTACGAGCGGCTCGGCTTCACCGTCACCGCCGACGTCGAGGTGCCCGAAGGACCGCGCACCTGGTGCATGACCCGCAAGCCCGGTGCCTGA  ATGCCGGCCCACTTGCTGCAGGACGATATCTCTAGCTCCTATACCACCACCACCACCATTACAGCGCCTCCCTCCAGGGTCCTGCAGAATGGAGGAGATAAGTTGGAGACGATGCCCCTCTACTTGGAAGACGACATTCGCCCTGATATAAAAGATGATATATATGACCCCACCTACAAGGATAAGGAAGGCCCAAGCCCCAAGGTTGAATATGTCTGGAGAAACATCATCCTTATGTCTCTGCTACACTTGGGAGCCCTGTATGGGATCACTTTGATTCCTACCTGCAAGTTCTACACCTGGCTTTGGGGGGTATTCTACTATTTTGTCAGTGCCCTGGGCATAACAGCAGGAGCTCATCGTCTGTGGAGCCACCGCTCTTACAAAGCTCGGCTGCCCCTACGGCTCTTTCTGATCATTGCCAACACAATGGCATTCCAGAATGATGTCTATGAATGGGCTCGTGACCACCGTGCCCACCACAAGTTTTCAGAAACACATGCTGATCCTCATAATTCCCGACGTGGCTTTTTCTTCTCTCACGTGGGTTGGCTGCTTGTGCGCAAACACCCAGCTGTCAAAGAGAAGGGGAGTACGCTAGACTTGTCTGACCTAGAAGCTGAGAAACTGGTGATGTTCCAGAGGAGGTACTACAAACCTGGCTTGCTGATGATGTGCTTCATCCTGCCCACGCTTGTGCCCTGGTATTTCTGGGGTGAAACTTTTCAAAACAGTGTGTTCGTTGCCACTTTCTTGCGATATGCTGTGGTGCTTAATGCCACCTGGCTGGTGAACAGTGCTGCCCACCTCTTCGGATATCGTCCTTATGACAAGAACATTAGCCCCCGGGAGAATATCCTGGTTTCACTTGGAGCTGTGGGTGAGGGCTTCCACAACTACCACCACTCCTTTCCCTATGACTACTCTGCCAGTGAGTACCGCTGGCACATCAACTTCACCACATTCTTCATTGATTGCATGGCCGCCCTCGGTCTGGCCTATGACCGGAAGAAAGTCTCCAAGGCCGCCATCTTGGCCAGGATTAAAAGAACCGGAGATGGAAACTACAAGAGTGGCTGA |

***Supplementary Table 2. Primer sequence***

|  | Forward primer (5′→3′) | Reverse primer (5′→3′) |
| --- | --- | --- |
| IGF2BP3  SCD  Pparg  Fabp4  Fasn  C/ebpα  METTL3  METTL14  β-actin  M^6^A-SCD | TATATCGGAAACCTCAGCGAGA  CAGGACGAUAUCUCUAGCUTT  TCGCTGATGCACTGCCTATG  AAGGTGAAGAGCATCATAACCCT  AGAGATCCCGAGACGCTTCT  GCGGGAACGCAACAACATC  TTGTCTCCAACCTTCCGTAGT  CTTTCAGAGAACAAAGGAACACTGCTCCCTCCATCCTGGCCTCGCTGT  AACCAGCTAGCGTGCAAGG | GGACCGAGTGCTCAACTTCT  AGCUAGAGAUAUCGUCCUGTT  GAGAGGTCCACAGAGCTGATT  TCACGCCTTTCATAACACATTCC  GCTTGGTCCTTTGAAGTCGAAGA  GTCACTGGTCAACTCCAGCAC  CCAGATCAGAGAGGTGGTGTAG  CTTCTACCAAGACAAAAATGCTCA  GTCACCTTCACCGTTCC  GCATCTTGGCTCTCGGATG |

***Supplementary Table 3. Primary and secondary antibodies and dilution ratio***

| Target  IGF2BP3  SCD  Pparg  Fabp4  Fasn  C/ebpα  METTL14  β-actin  anti-mouse HRP  secondary antibody  anti-rabbit HRP  secondary antibody | Company  Proteintech,China  Bioss,USA  Proteintech,China  Proteintech,China  Proteintech,China  Proteintech,China  Proteintech,China  Proteintech,China  Immunoway,USA  Immunoway,USA | Cat.No.  14642-1-AP  Bs-3787R  16643-1-AP  12802-1-AP  10624-2-AP  18311-1-AP  26158-1-AP  60008-1-Ig  RS0001  RS0002 | Dilution ratio  1:5000  1:1000  1:5000  1:20000  1:20000  1:1000  1:2000  1:5000  1:5000  1:5000 |
| --- | --- | --- | --- |


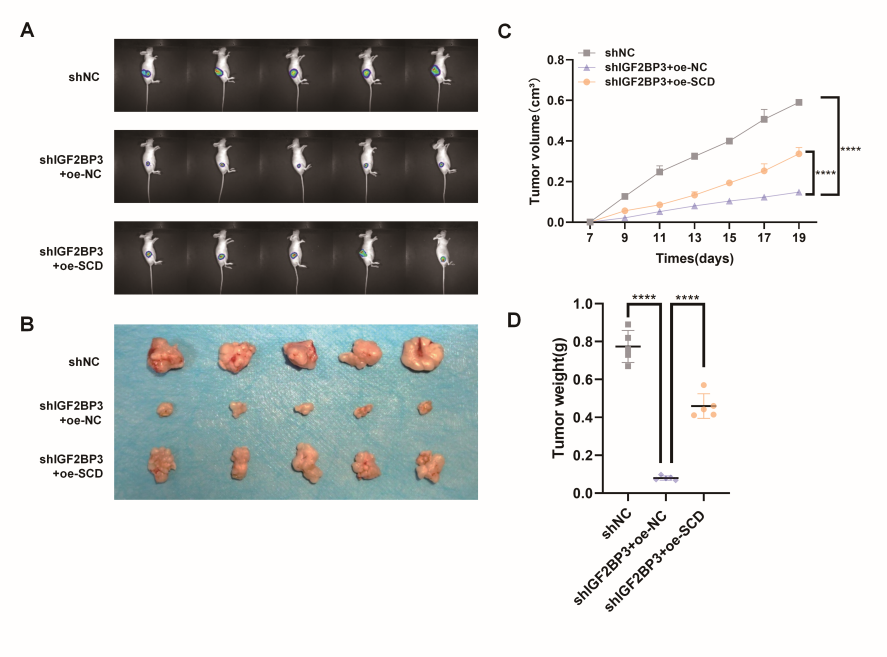


**Figure S. IGF2BP3 enhances the growth of CC tumor in vivo.**

**(A, B)** Siha cells stably expressing shNC, shIGF2BP3,or shIGF2BP3+oe-SCD were subcutaneously injected into the right back of nude mice. Tumor images were captured using the PerkinElmer IVIS preclinical in vivo imaging system 20 days **(A)** and 30 days **(B)** after injection. **(C)** The tumor volumes were measured every two days from the 7th day after Siha cells injection. **(D)** The weights of transplanted tumors were measured.
