# Supplementary material for: IGF2BP3 enhances lipid metabolism in cervical cancer by upregulating the expression of SCD
Source: Cell Death Dis. 2024 Feb 14;15(2):138. doi: 10.1038/s41419-024-06520-0 (PMC10867090; doi:10.1038/s41419-024-06520-0)

Figure 2B-Hela-ACTIN

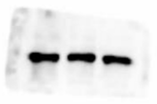

Figure 2B-Hela-IGF2BP3

Figure 2B-Siha-ACTIN

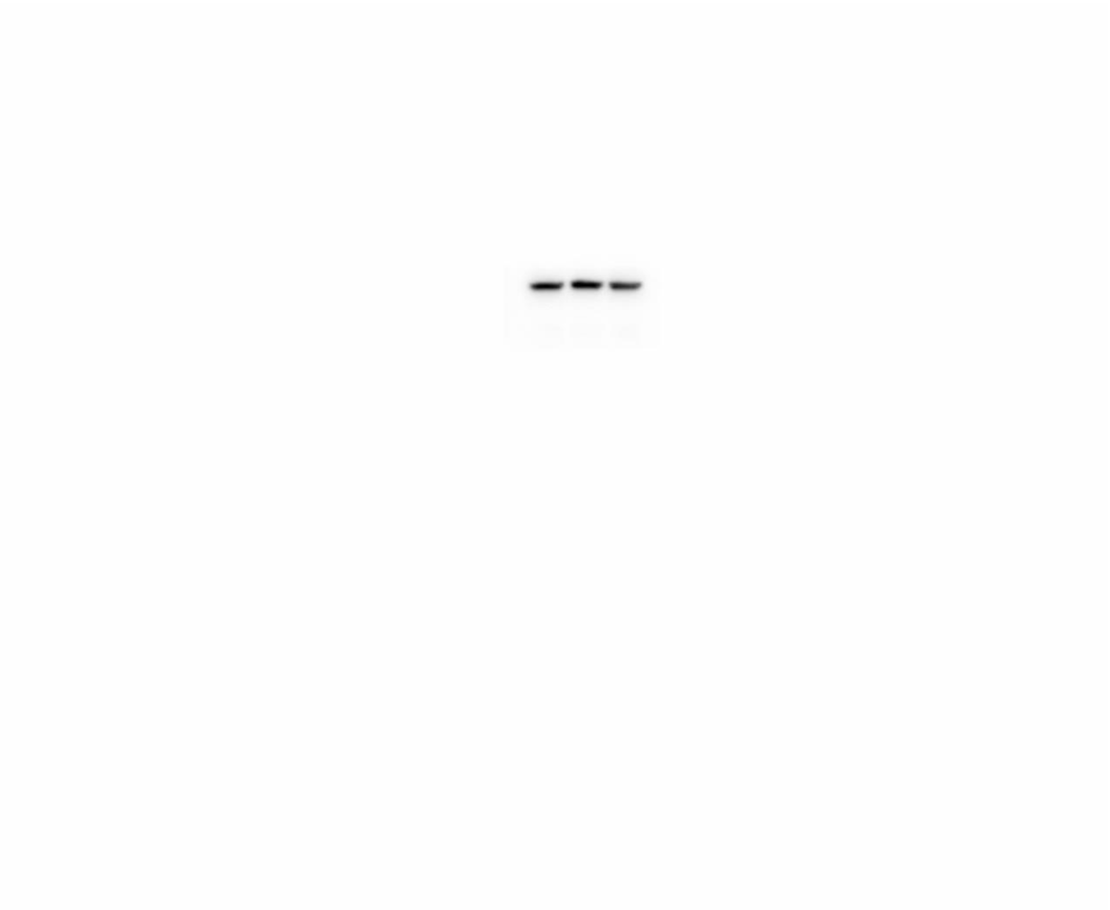

Figure 2B-Siha-IGF2BP3

Figure 3I-Hela-ACTIN

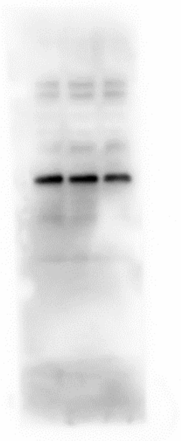

Figure 3I-Hela-Cebpa

Figure 3I-Hela-Fabp4

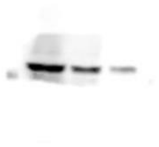

Figure 3I-Hela-Fasn

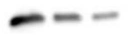

Figure 3I-Hela-IGF2BP3

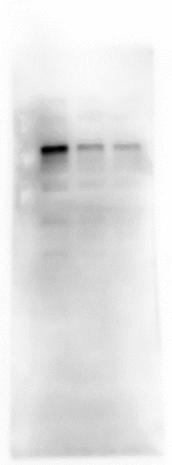

Figure 3I-Hela-Pparg

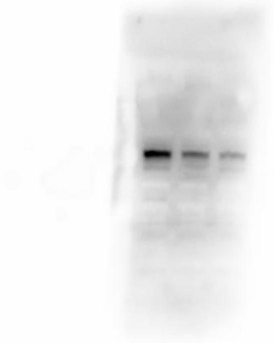

Figure 3I-Hela-SCD

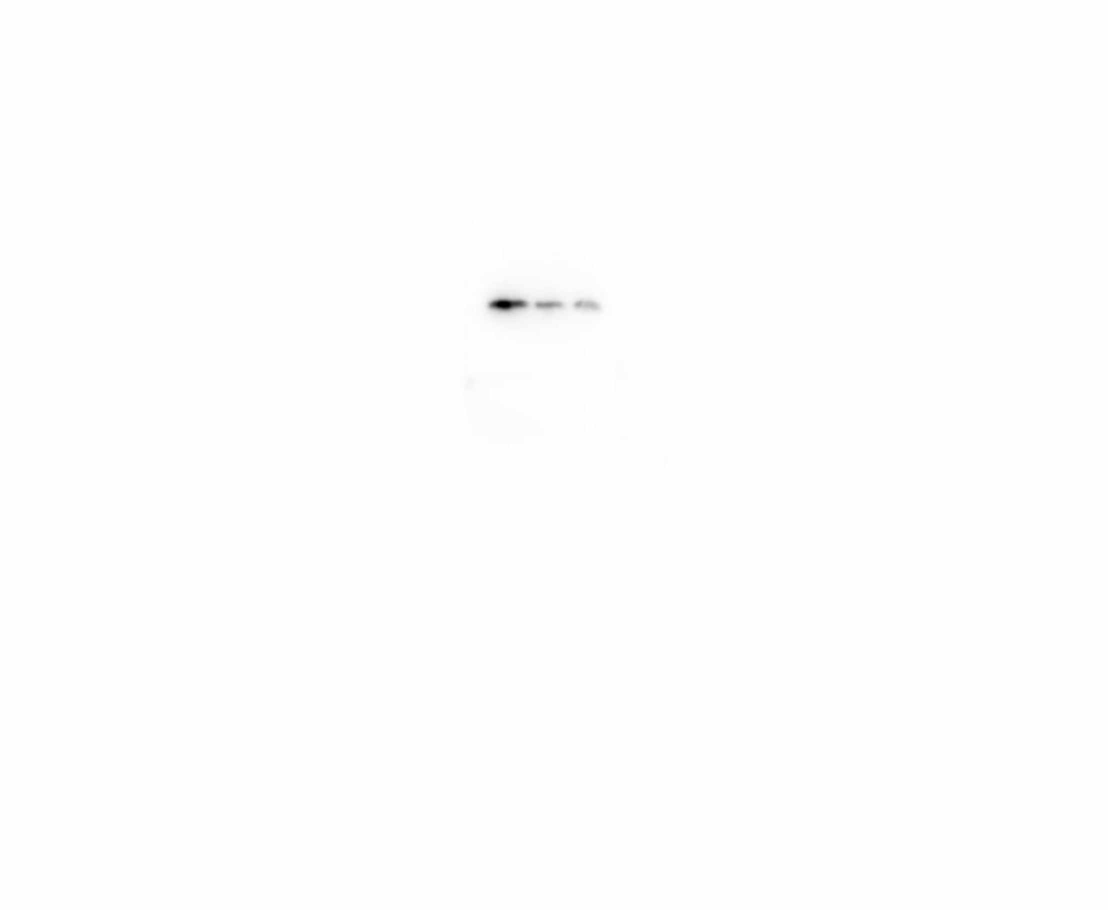

Figure 3I-Siha-ACTIN

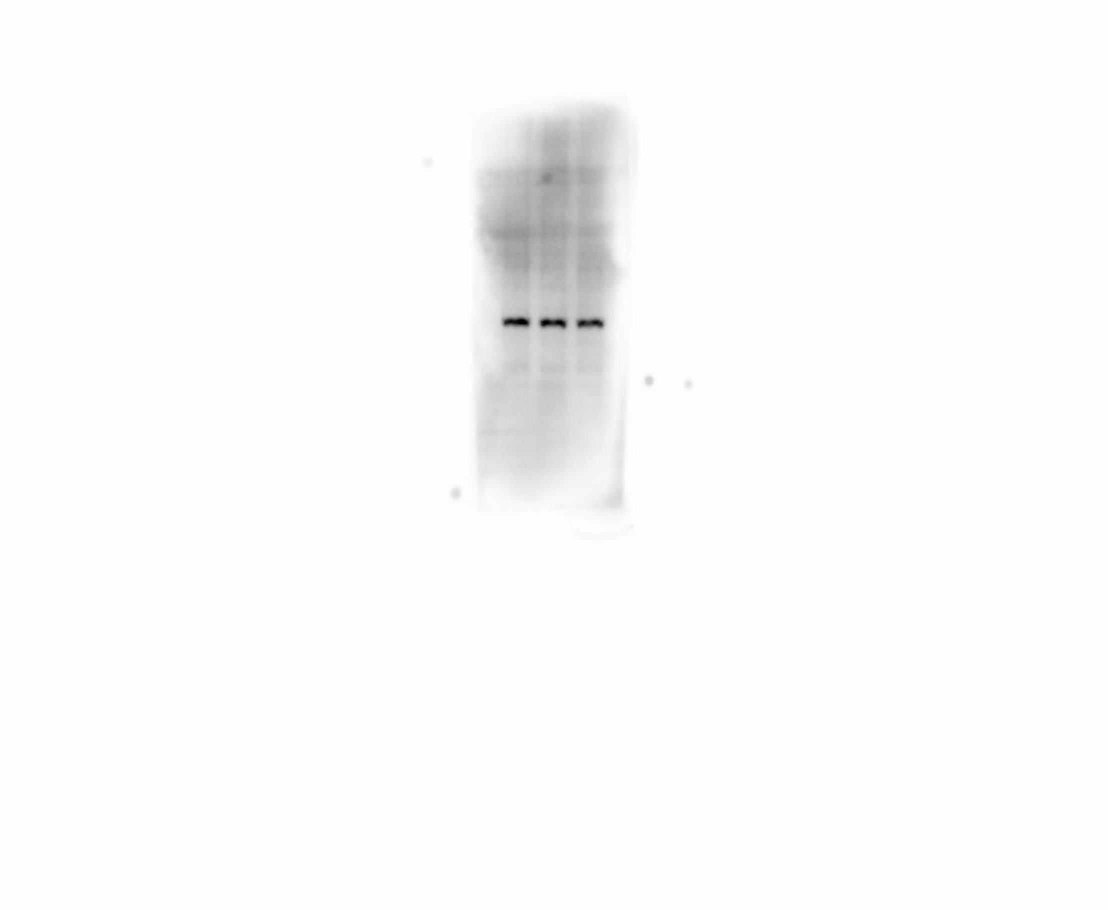

Figure 3I-Siha-Cebpa

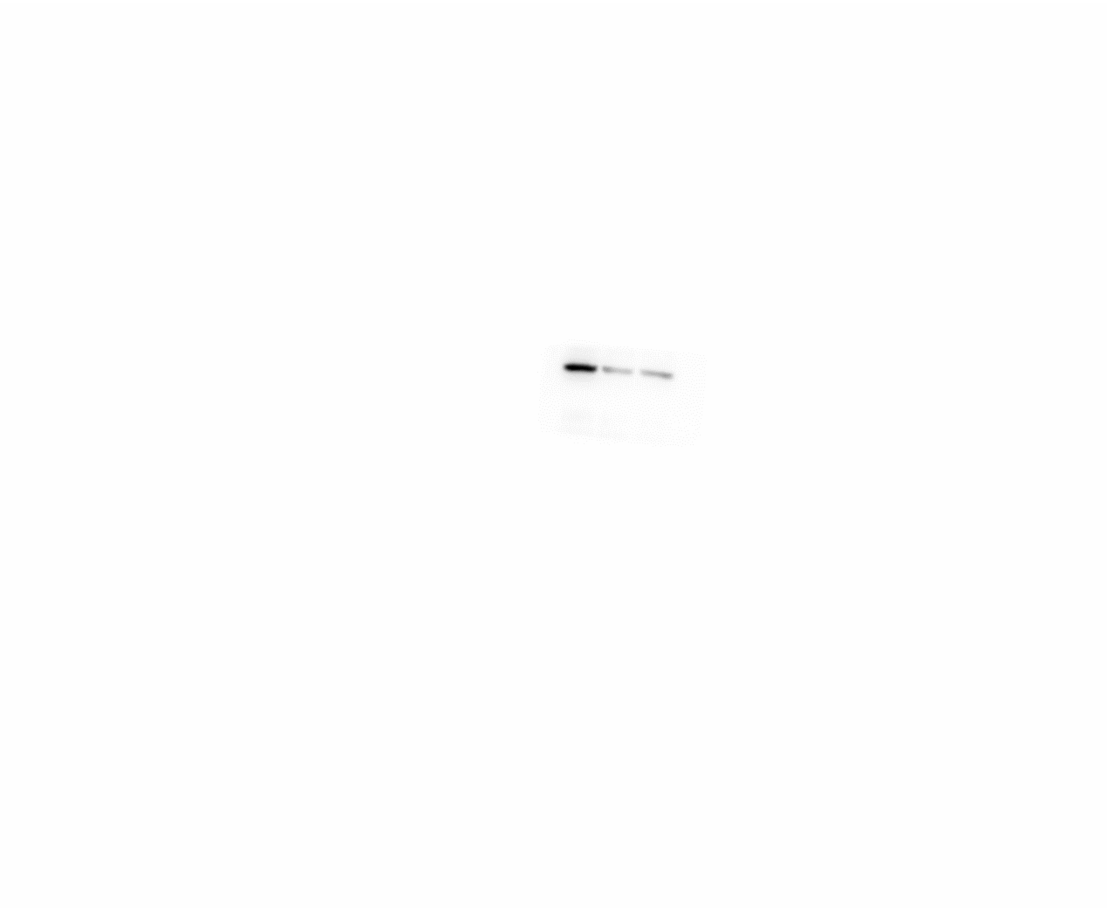

Figure 3I-Siha-Fabp4

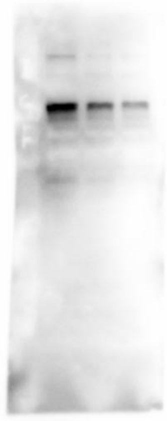

Figure 3I-Siha-Fasn

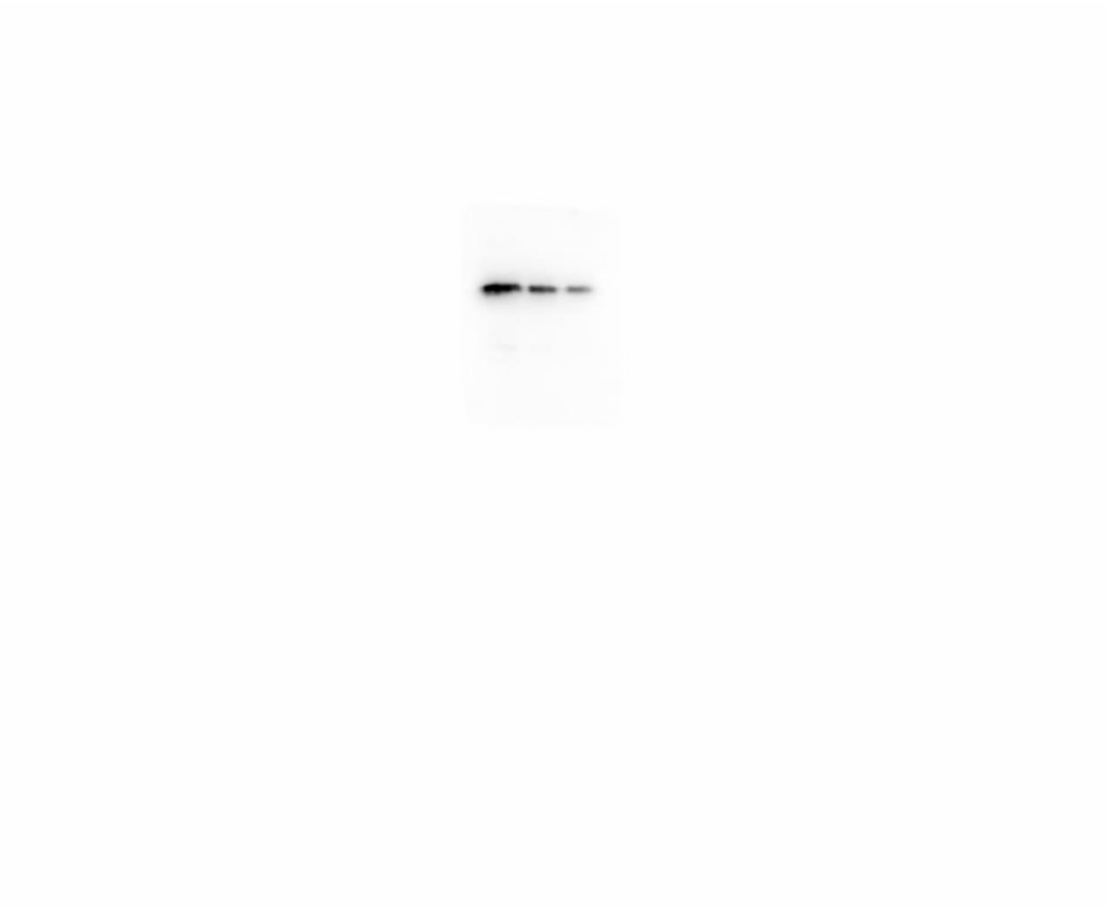

Figure 3I-Siha-IGF2BP3

Figure 3I-Siha-Pparg

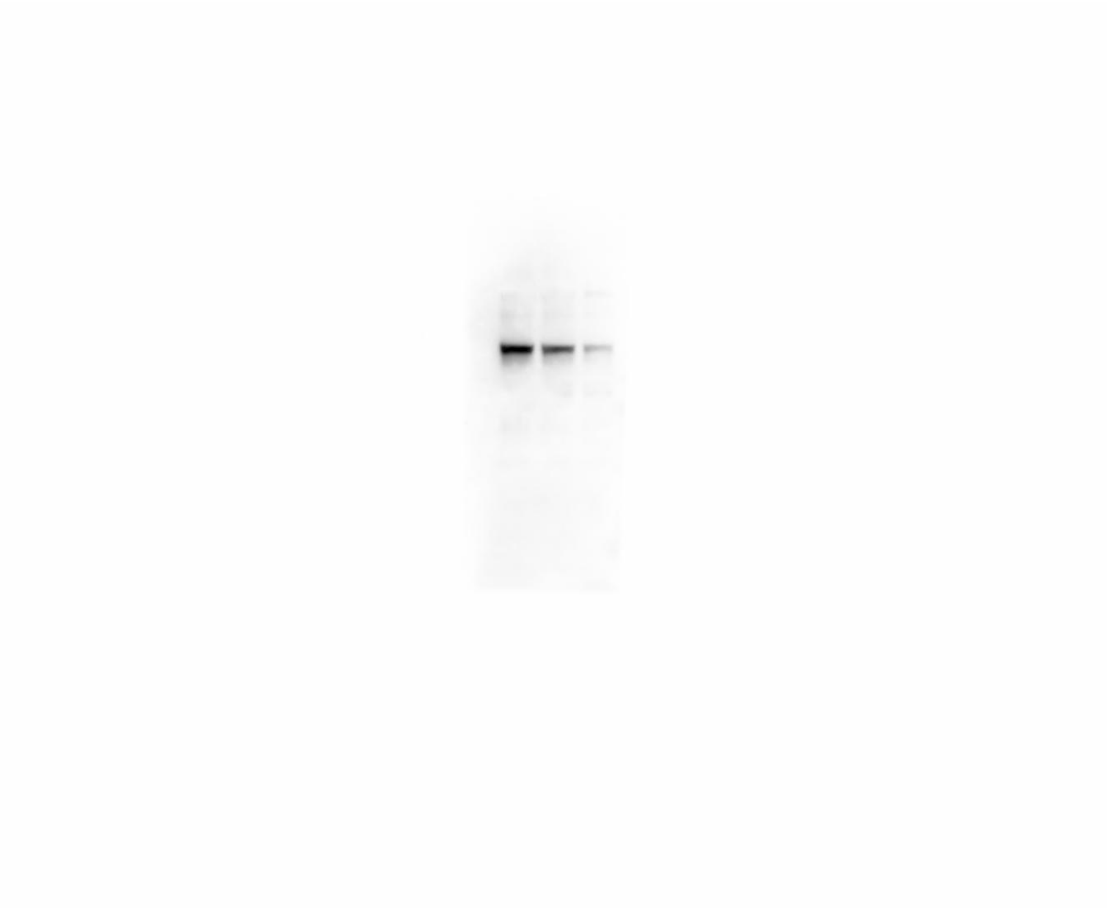

Figure 3I-Siha-SCD

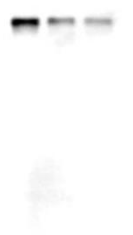

Figure 4H-Hela-ACTIN

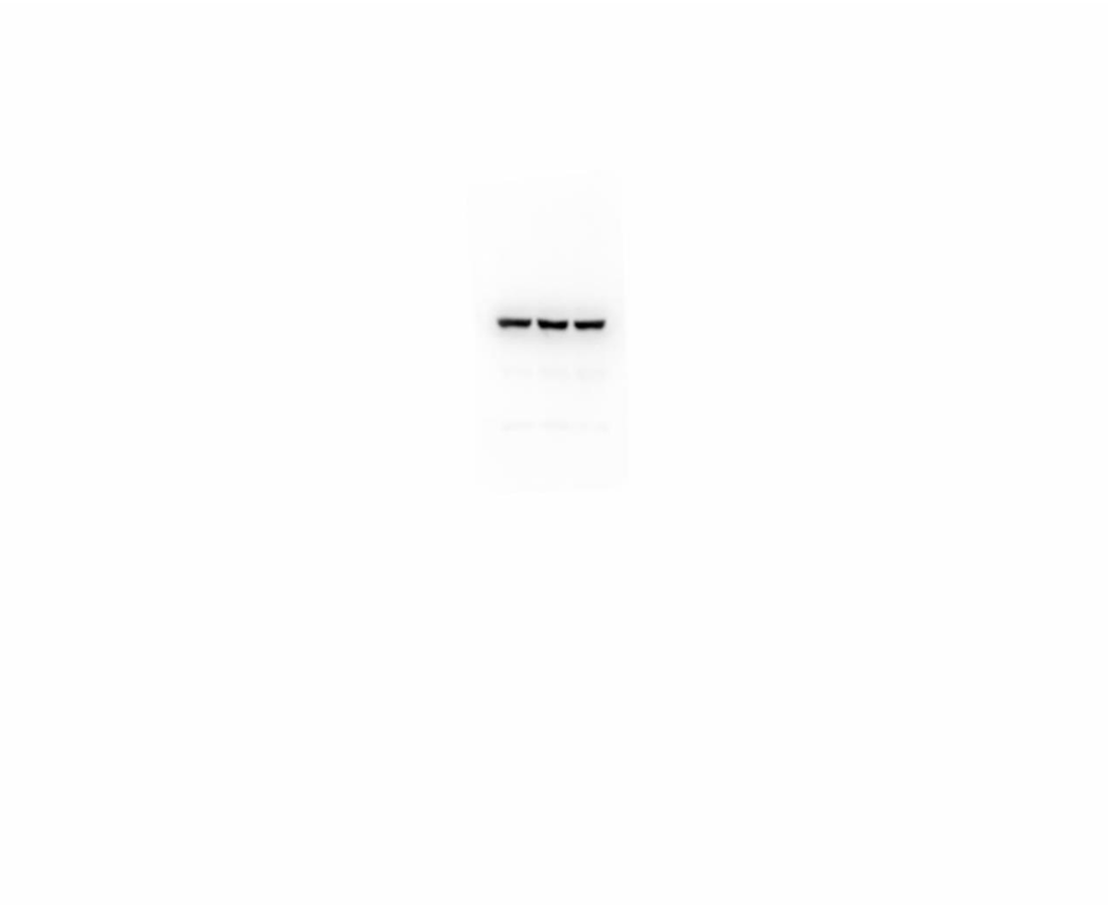

Figure 4H-Hela-Cebp4

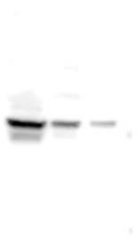

Figure 4H-Hela-Fabp4

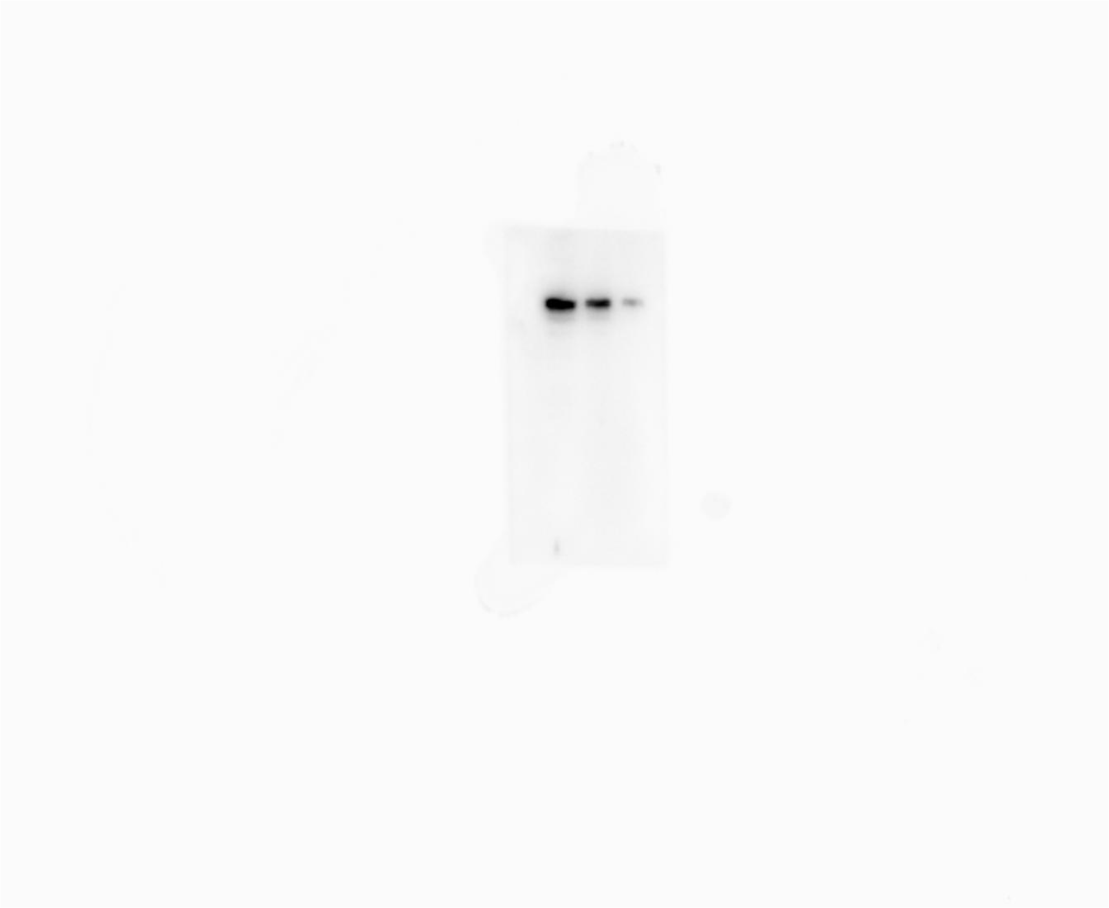

Figure 4H-Hela-Fasn

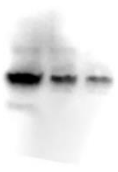

Figure 4H-Hela-Met

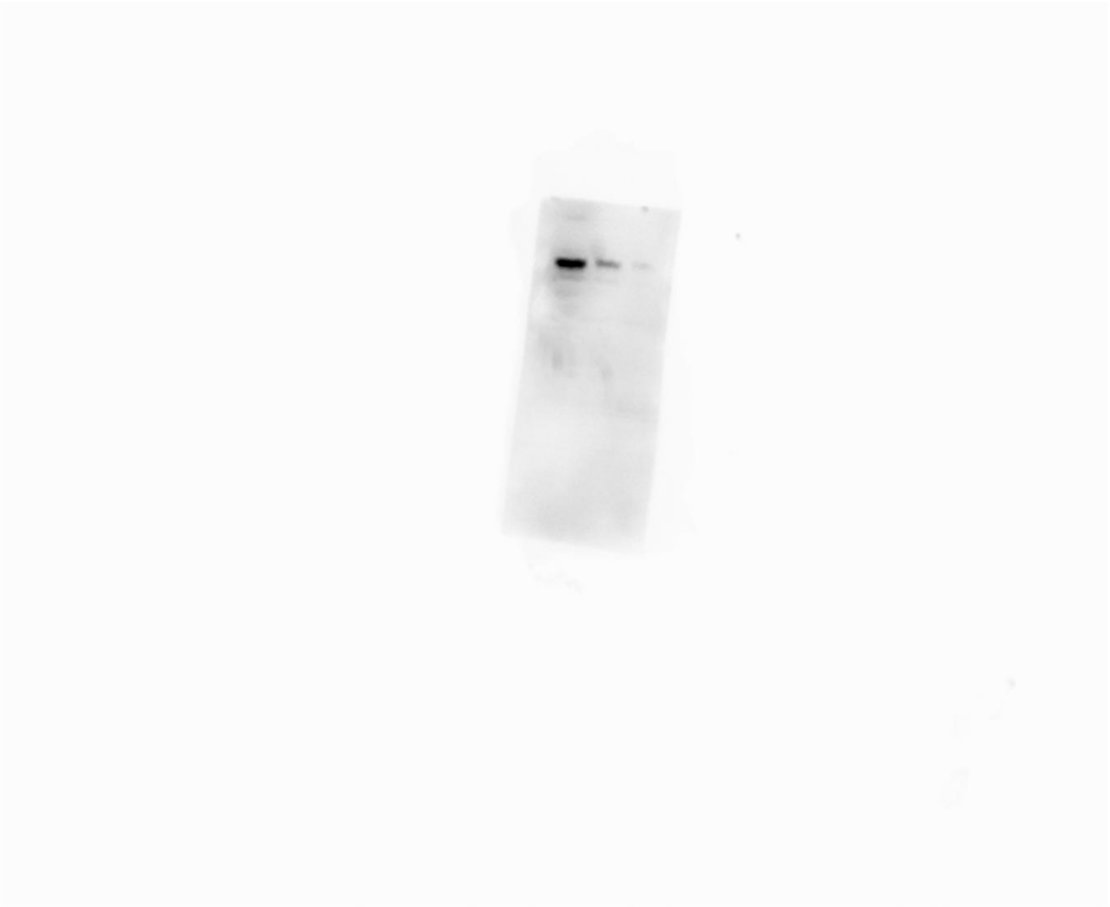

Figure 4H-Hela-Pparg

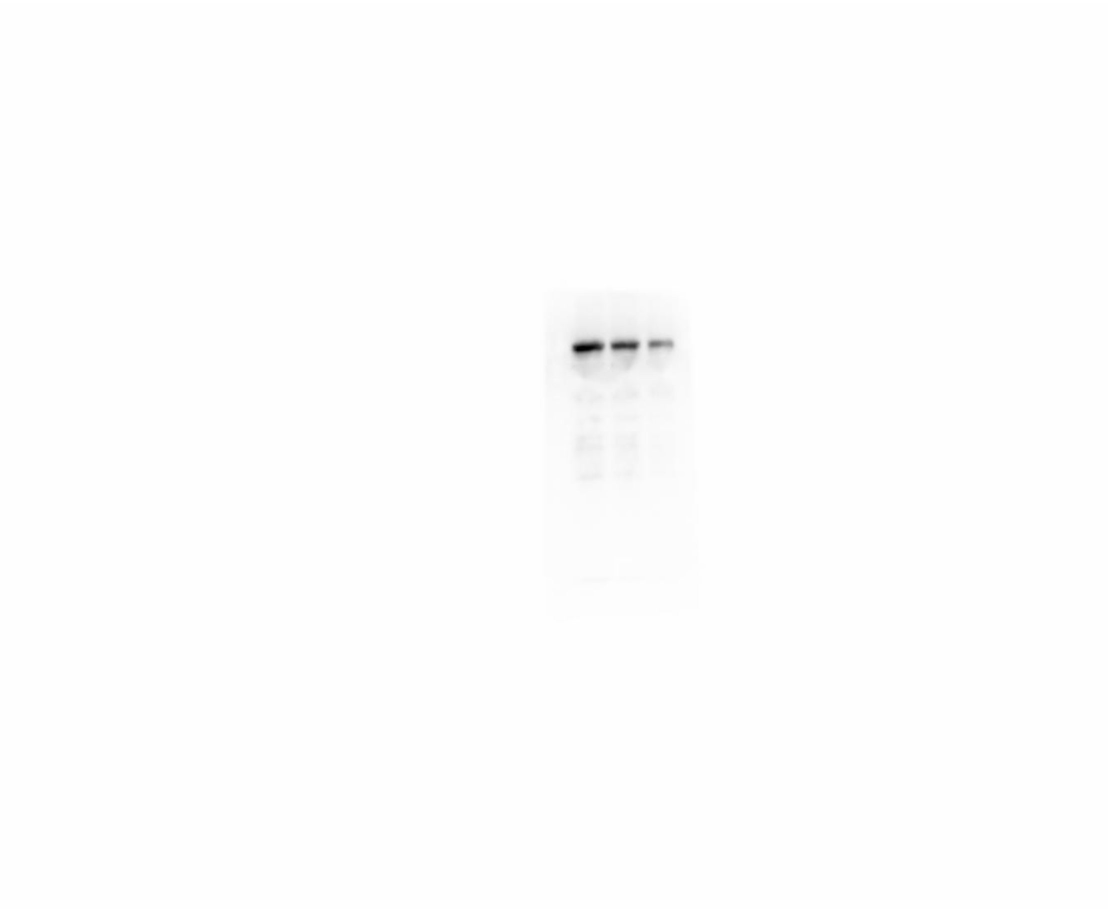

Figure 4H-Hela-SCD

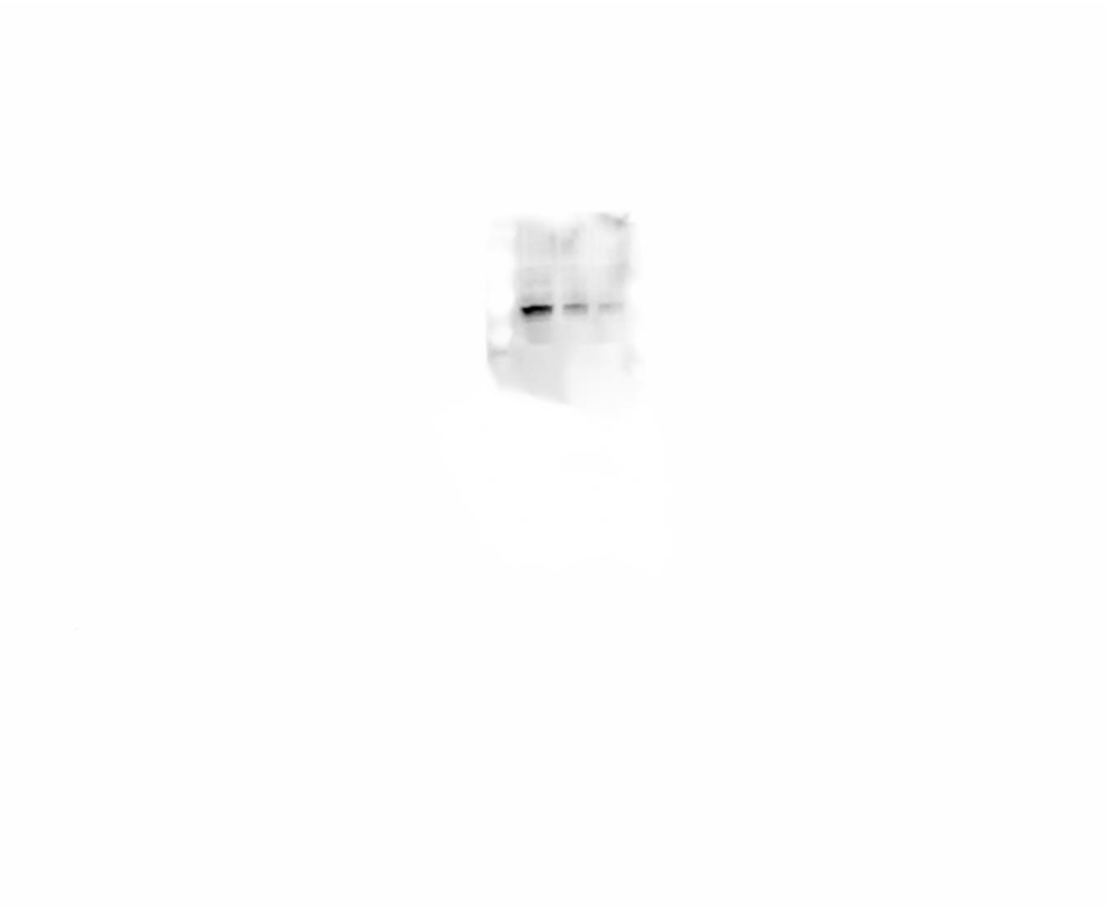

Figure 4H-Siha-ACTIN

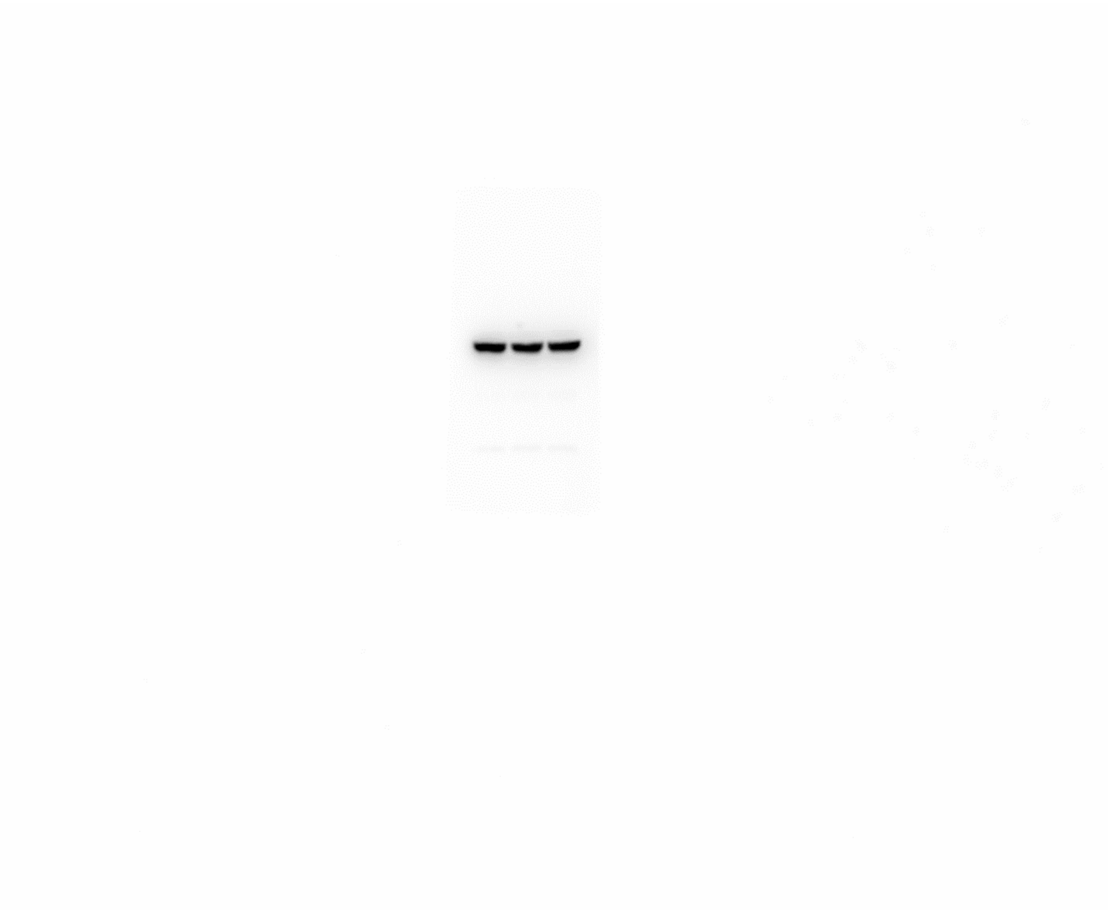

Figure 4H-Siha-Cebp4

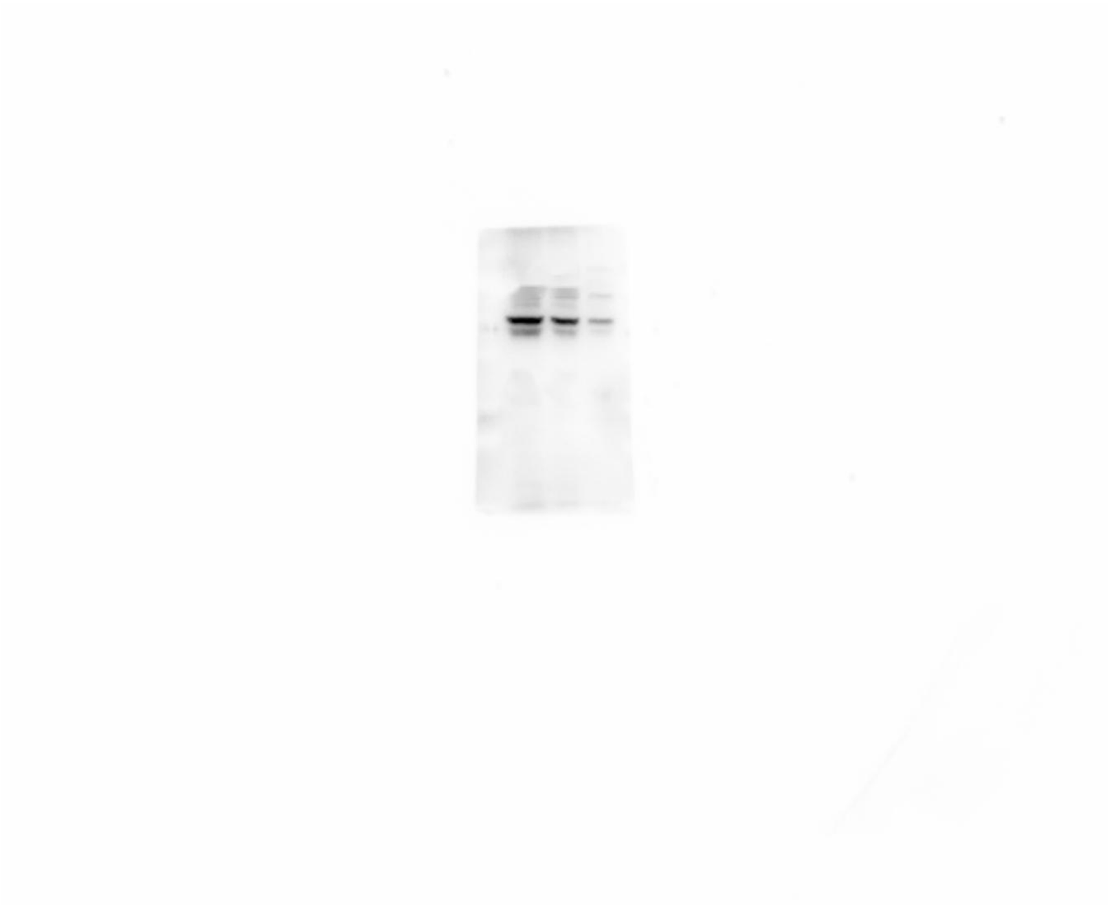

Figure 4H-Siha-Fabp4

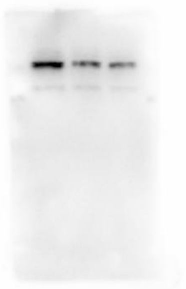

Figure 4H-Siha-Fasn

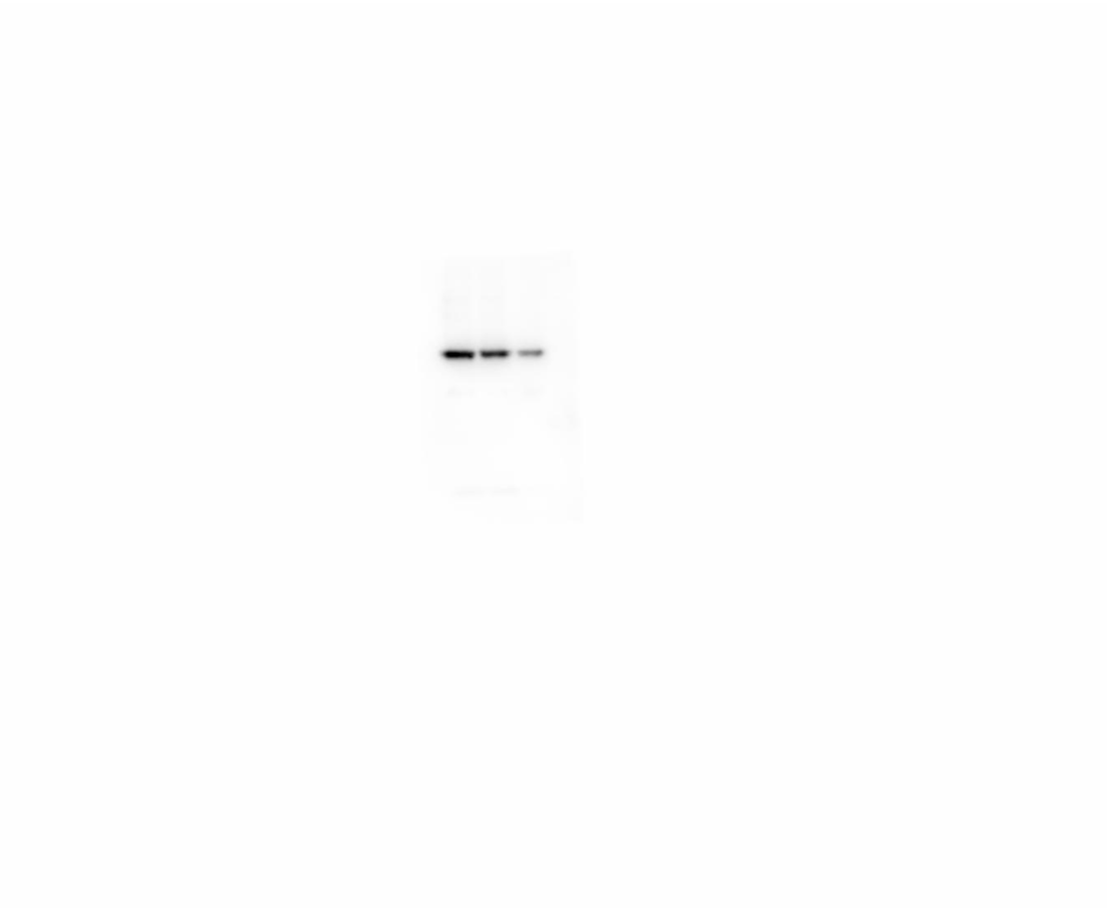

Figure 4H-Siha-Met

Figure 4H-Siha-Pparg

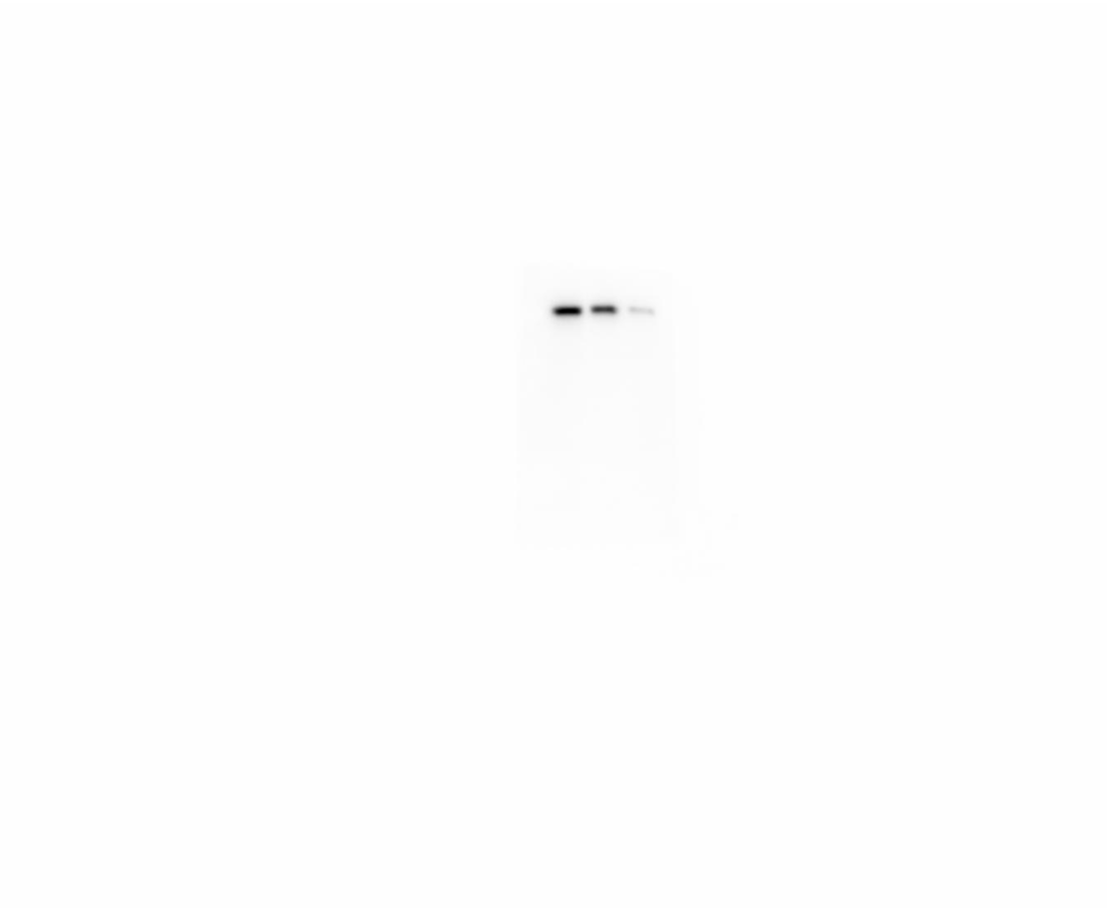

Figure 4H-Siha-SCD

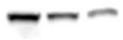

Figure 4I-input

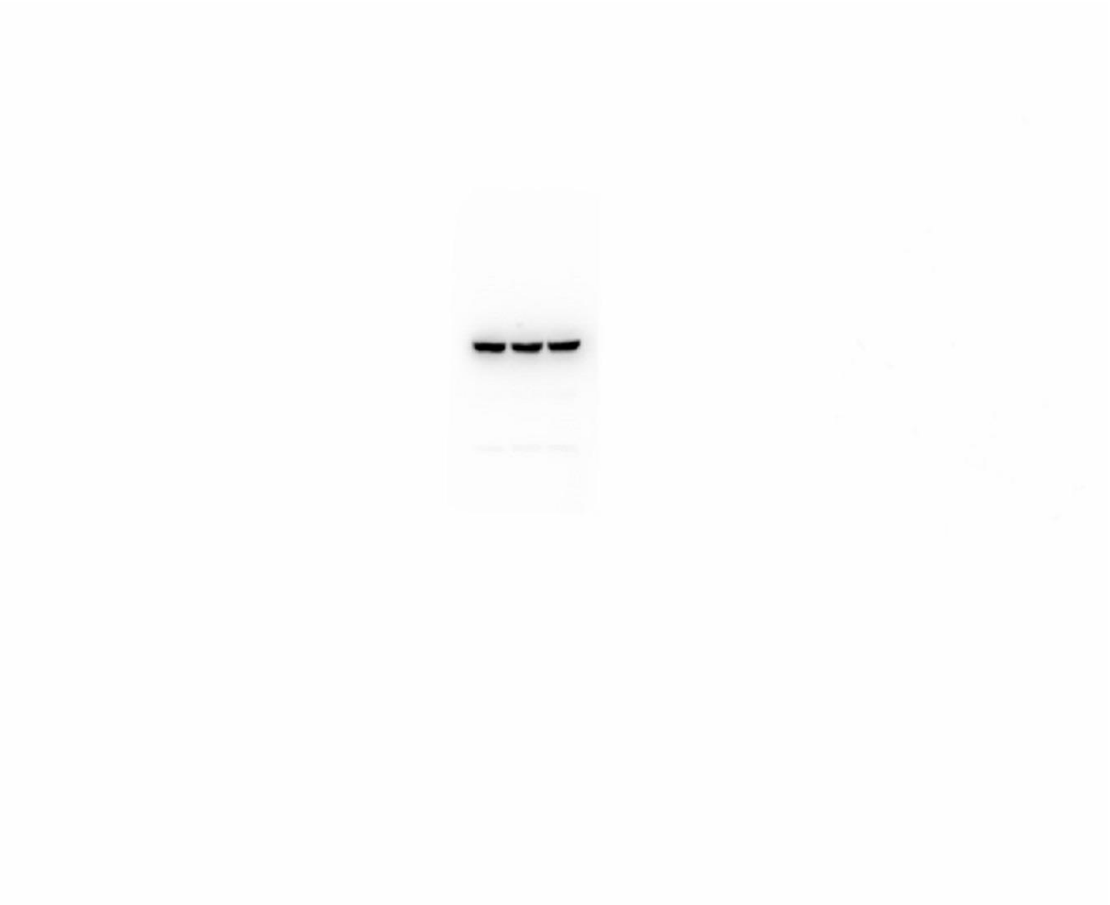

Figure 4I-IP

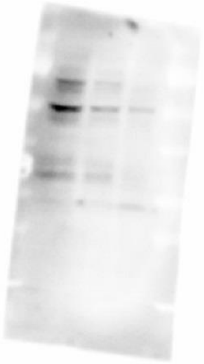

Figure 5B-Hela ACTIN

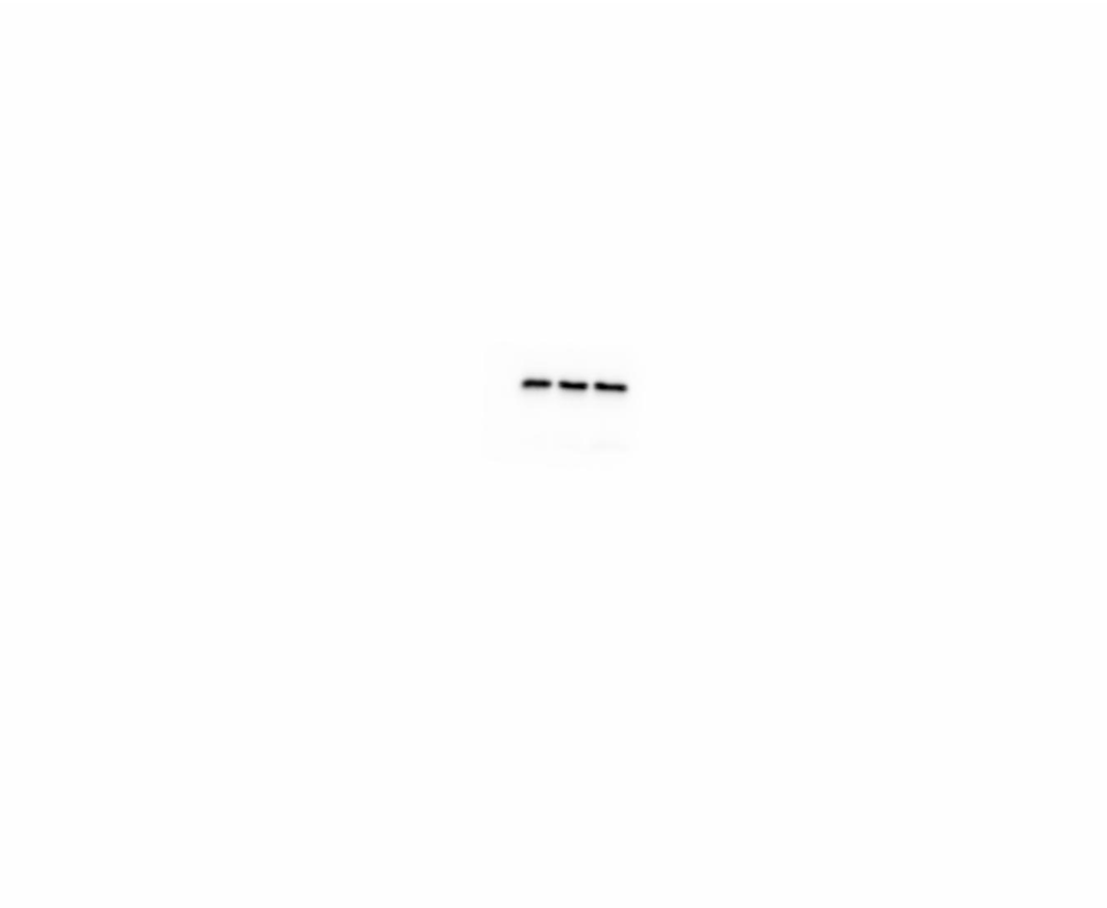

Figure 5B-Hela-Cebpa

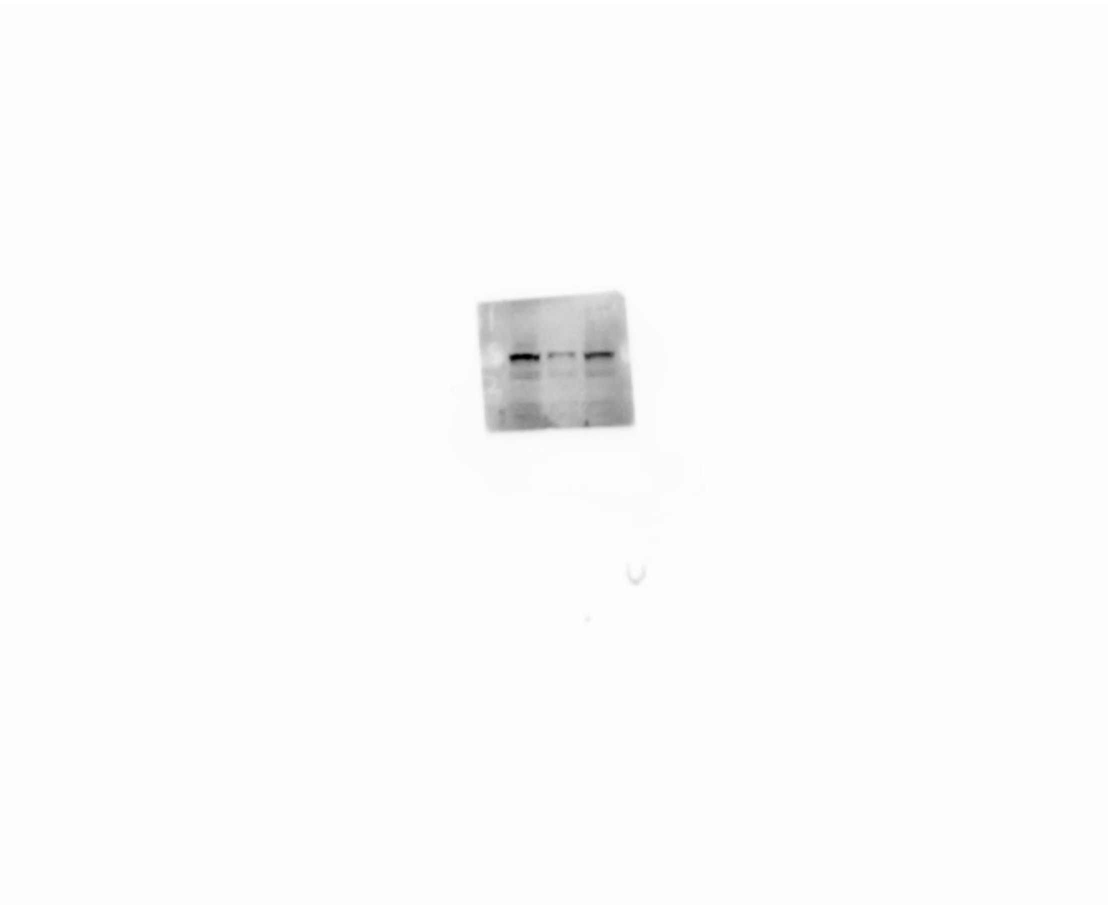

Figure 5B-Hela-Fabp4

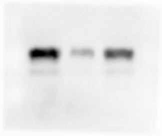

Figure 5B-Hela-Fasn

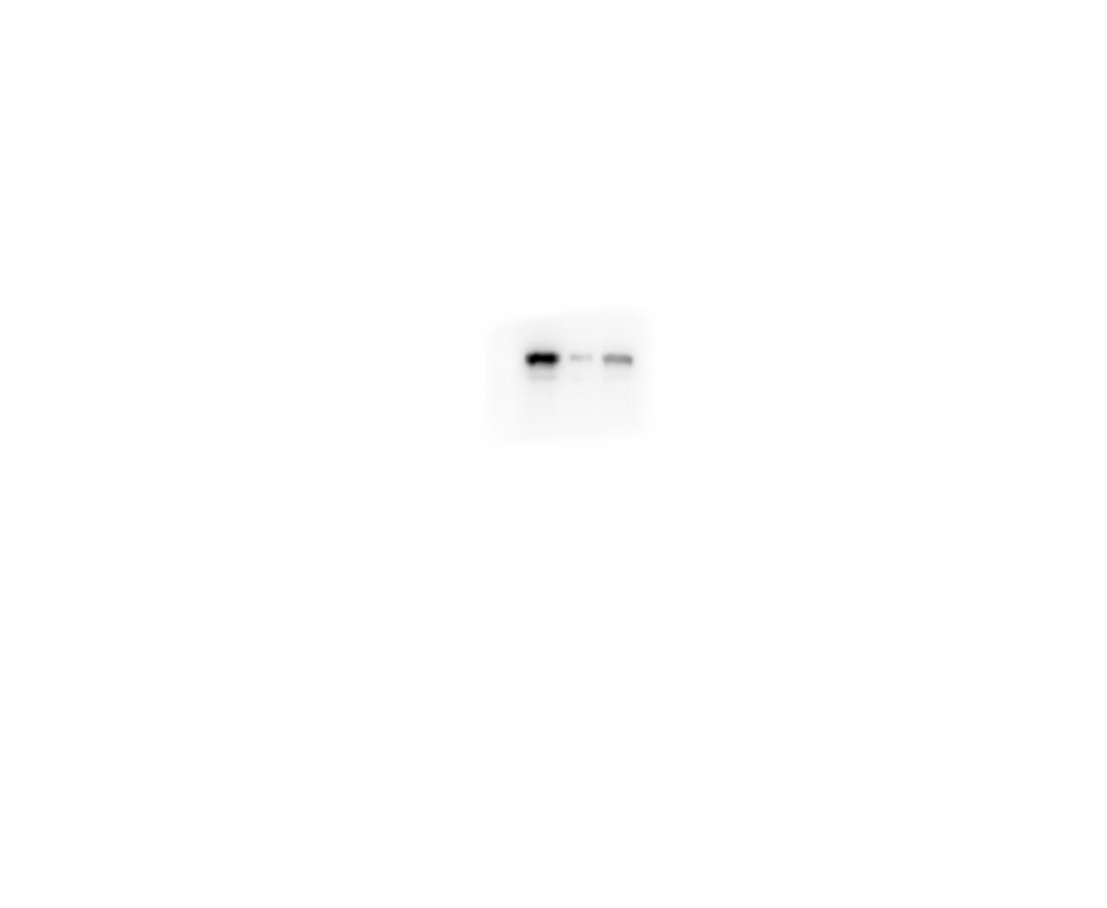

Figure 5B-Hela-IGF2BP3

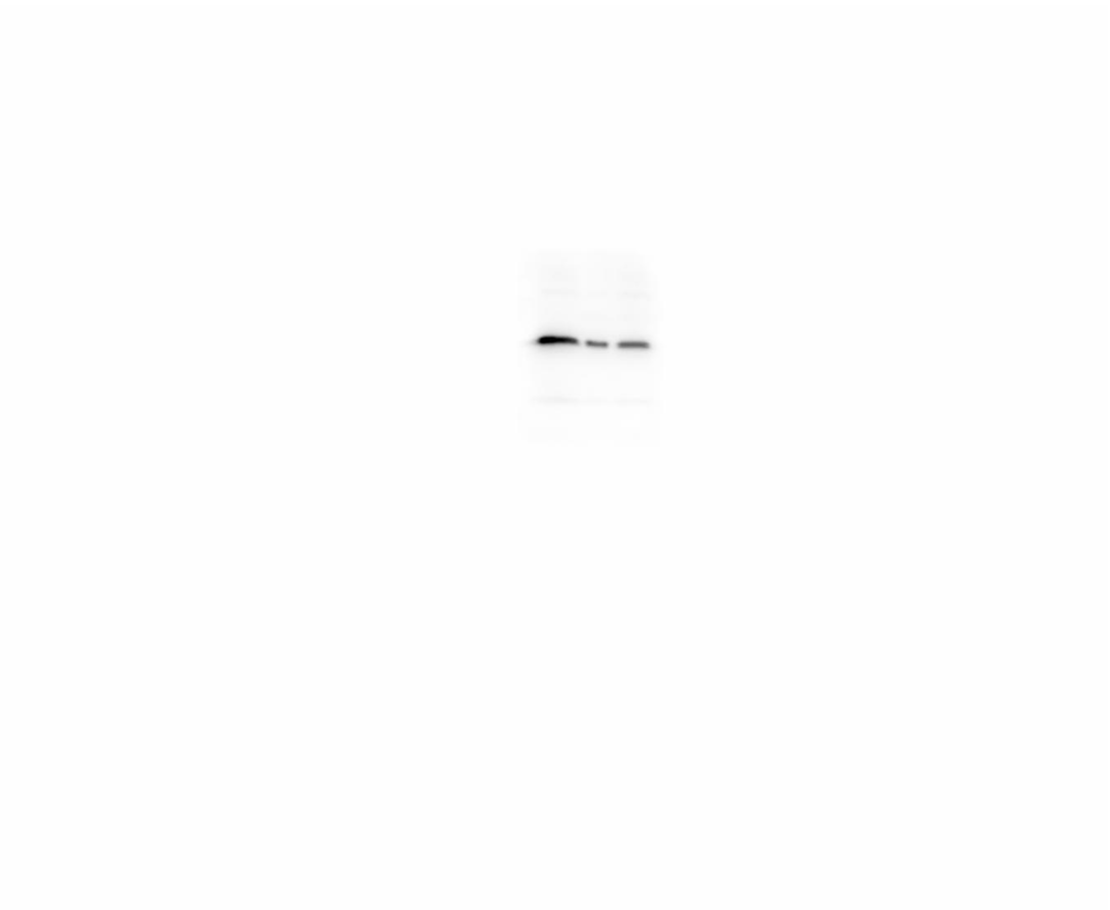

Figure 5B-Hela-Pparg

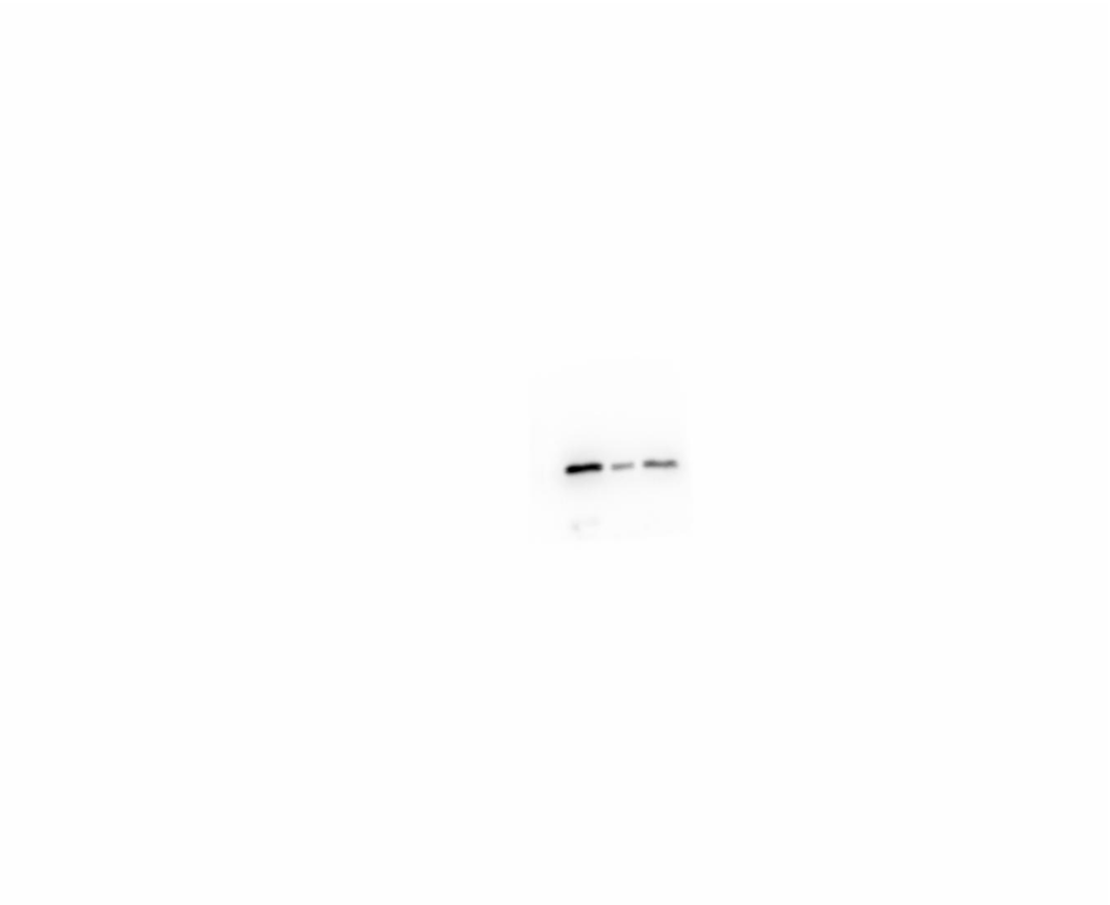

Figure 5B-Hela-SCD

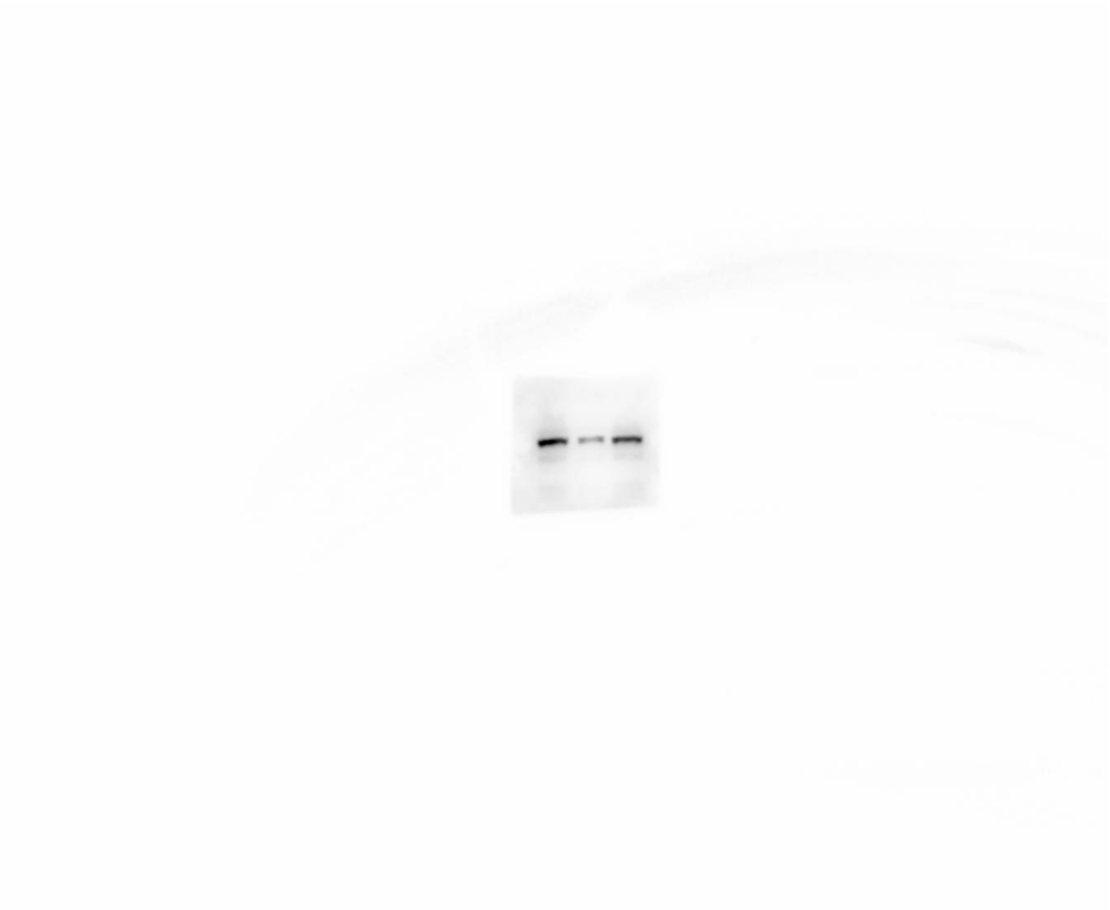

Figure 5B-Siha ACTIN

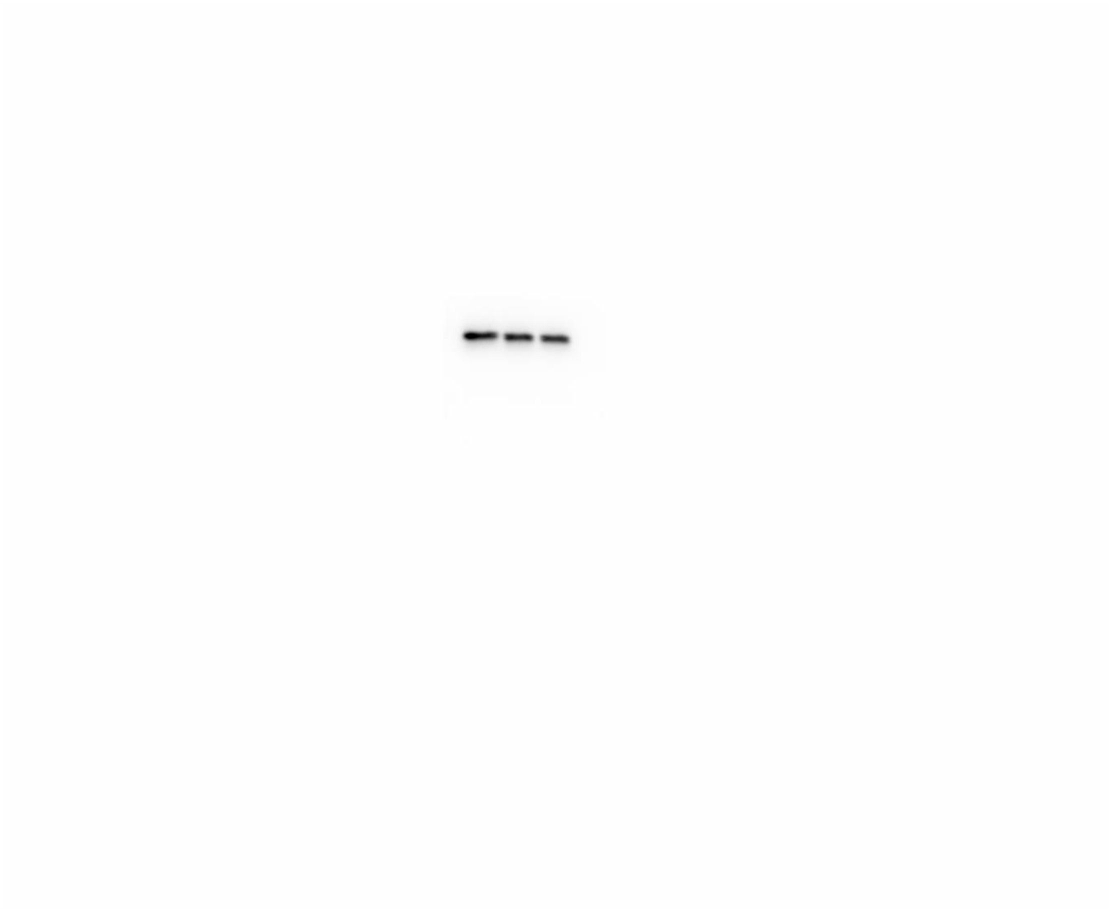

Figure 5B-Siha-Cebpa

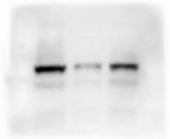

Figure 5B-Siha-Fabp4

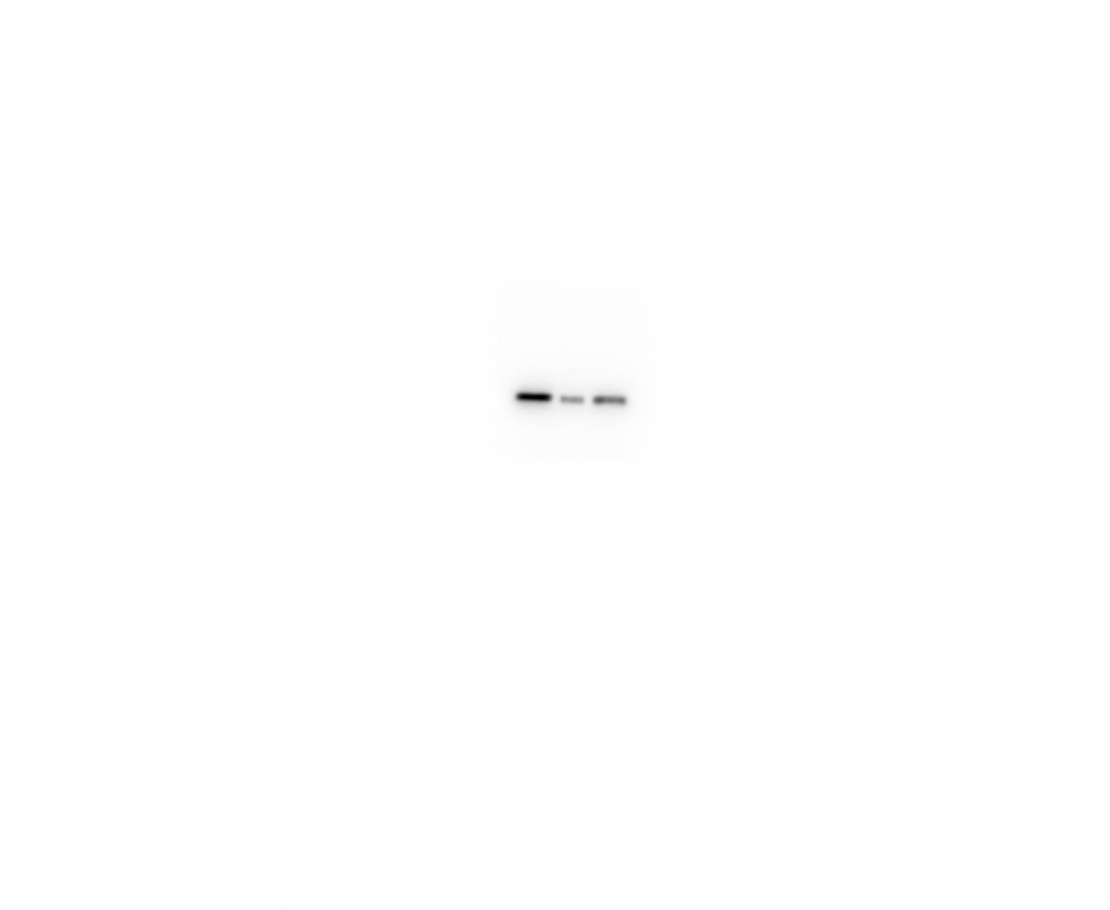

Figure 5B-Siha-Fasn

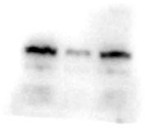

Figure 5B-Siha-IGF2BP3

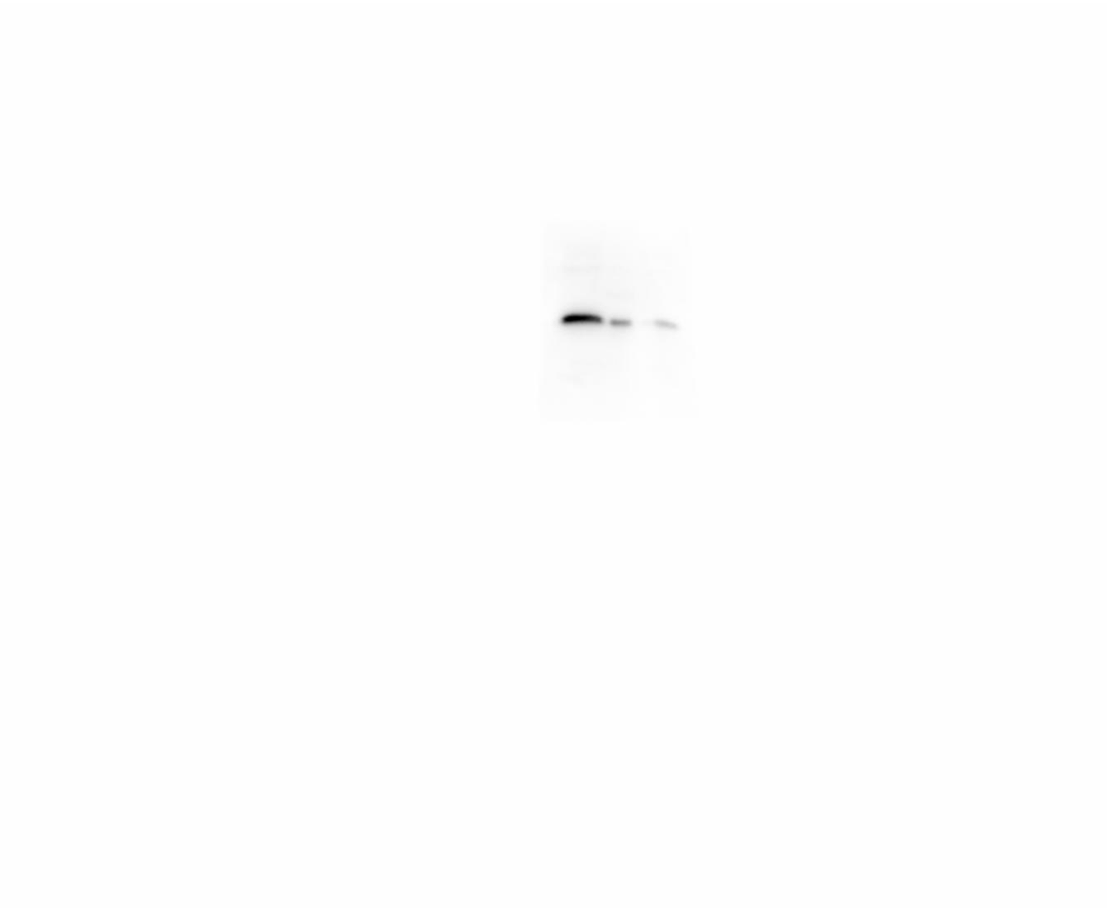

Figure 5B-Siha-Pparg

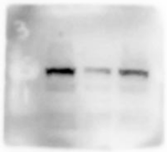

Figure 5B-Siha-SCD

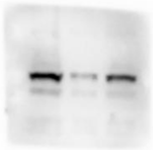

Supplement: Supplementary file 2 — Supplemental Material-western [file 41419_2024_6520_MOESM2_ESM.pdf]
